# Fabrication of Various Plasmonic Pt Nanostructures via Indium Assisted Solid-State Dewetting: From Small Nanoparticles to Widely Connected Networks

**DOI:** 10.3390/nano9060831

**Published:** 2019-05-31

**Authors:** Sanchaya Pandit, Mao Sui, Sundar Kunwar, Puran Pandey, Sandesh Pant, Jihoon Lee

**Affiliations:** 1 Department of Electronic Engineering, College of Electronics and Information, Kwangwoon University, Nowon-gu, Seoul 01897, Korea; sanchaya7@gmail.com (S.P.); kunwarankees23@gmail.com (S.K.); ppcpurans@gmail.com (P.P.); sandeshpant2@gmail.com (S.P.); 2 Institute of Hybrid Materials, College of Materials Science and Engineering, Qingdao University, Qingdao 266071, China

**Keywords:** platinum (Pt) nanoparticles, solid state dewetting, in sublimation, In-Pt bilayers, localized surface plasmon resonance

## Abstract

In this paper, the modified solid-state dewetting (MSSD) of well-defined and various uniform Pt nanostructures is demonstrated by the auxiliary diffusion enhancement. The MSSD utilizes the introduction of metallic indium (In) layers with high diffusivity in between sapphire and platinum (Pt) layer, through which the global diffusion and dewetting of metallic atoms can be significantly enhanced. Subsequently, the In atoms can be sublimated from the NP matrix, resulting in the formation of pure Pt NPs. By the systematic control of In and Pt bi-layer thickness, various areal density, size and configuration of Pt NPs are demonstrated. The In_2 nm_/Pt_2 nm_ bilayers establish very small and highly dense NPs throughout the temperature range due to the early maturation of growth. Intermediate size of NPs is demonstrated with the In_45 nm_/Pt_15 nm_ bilayers with the much improved interparticle spacings by annealing between 650 and 900 °C for 450 s. Finally, the In_30 nm_/Pt_30 nm_ bilayers demonstrate the widely connected network-like nanostructures. In addition, the finite difference time domain (FDTD) simulation is employed to exploit the local electric field distributions at resonance wavelengths. The dewetting characteristics of In/Pt bilayers is systematically controlled by the modifications of layer thickness and annealing temperature and is systematically described based on the diffusion of atoms, Rayleigh instability and surface energy minimization mechanism. The optical properties demonstrate dynamic and widely tunable localized surface plasmon resonance (LSPR) responses depending upon the various surface morphologies of Pt nanostructures.

## 1. Introduction

Metallic nanoparticles (NPs) have demonstrated valuable tunable plasmonic, optical, electrical and catalytic properties [1,2] and are essential components in various applications such as sensors [3], solar cells [4,5], bio-medicals [6] and catalysts [7]. For example, the incorporation of metallic NPs can significantly improve the light conversion efficiency of solar cells due to the enhanced light absorption in the visible range of solar radiation by the localized surface plasmon resonance (LSPR) [8]. The LSPR corresponds to the collective oscillation of free electrons on the metallic NPs by the photon excitation and can be systematically tuned by the control of size, configuration and spacing of metallic NPs [9,10,11]. The plasmon resonance of noble metallic NPs such as silver, gold, copper and aluminum have attracted large scientific attentions in the energy and environment [12], water distillation [13], microscopy, membrane technology [14], nanomedicine [15], photonics and sensing fields. Among various metallic NPs, the Pt NPs are generally catalytically active systems for various chemical reactions due to the enhanced catalytic properties [16,17]. Specifically, the Pt catalysts have demonstrated high reactivity, inertness, stability on substrate and high hydrogen adsorption-desorption capability, which makes it unique material in the redox reactions, hydrogenation-dehydrogenation of compounds and electrocatalytic reactions for hydrogen evolution [18,19,20].

In addition, recently, few studies have evidenced the LSPR bands of Pt NPs in the UV-Vis-NIR wavelength [21,22]. Although the plasmon intensity of Pt NP is relatively weaker as compared to the Ag and Au, it can still offer large enhancement in the photocatalytic reactions [23,24,25]. The LSPR of Pt NPs can be improved and tuned in a wide range of wavelength via the fabrication of various surface morphology, size and configurations, which directly can affect the chemical reactivity and thus performance of photocatalytic applications [12,13,20]. The Pt nano-catalysts have been usually synthesized by the wet chemistry methods in the form of colloidal solutions and then dispersed onto a substrate using solvents, which could lead weak binding to the substrate, impurity and poor durability [14,21]. Meanwhile, the solid state dewetting (SSD) approach can provide highly stable, pure and large-scale fabrication of various Pt NPs on the solid substrate. However, due to the low diffusion coefficient of Pt atoms, the conventional SSD of Pt films generally yields widely connected or poor uniformity of Pt nanostructures even at very high temperatures [26,27,28,29]. The usage of sacrificial In layer between the Pt film and sapphire was previously demonstrated to enhance the dewetting with the fixed Pt layers thickness (10 nm). This study was focused on the effect of In layer demonstration and compared it with the pure Pt dewetting [30]. In addition, the effect of thickness ratios (1:3, 1:1 and 3:1) of In/Pt bilayers on dewetting behavior was been reported in another report [31]. The results signify an efficient route to improve and tune the size, density and configuration of Pt nanostructures at usual growth conditions.

**Figure 1:**
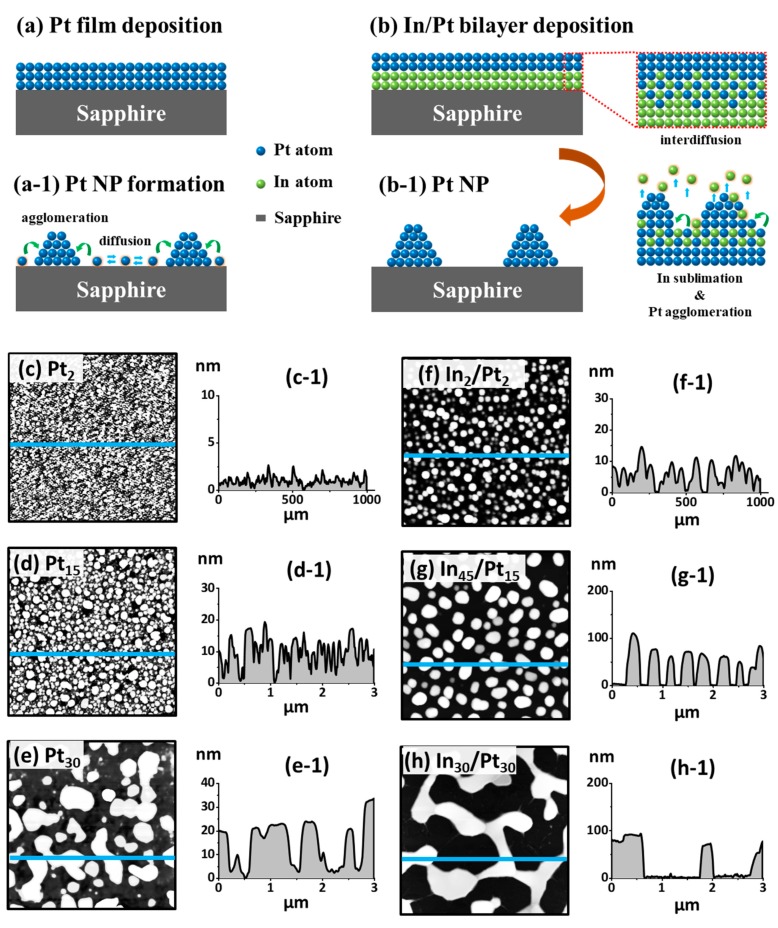
Comparison of the conventional solid state dewetting (SSD) and modified SSD of Pt film and In/Pt bilayer on sapphire. (**a**)–(**a-1**) Schematic of Pt nanoparticles (NPs) formation by the annealing of pure Pt thin film: i.e., conventional SSD. (**b**)–(**b-1**) Scheme of improved Pt NP fabrication with the In/Pt bilayer films: i.e., modified SSD. (**c**)–(**e**) AFM images of the Pt NPs fabricated with 2, 15 and 30 nm thick Pt films at 900 °C for 450 s. (**c-1**)–(**e-1**) Cross-sectional line profiles of Pt NPs in (**c**)–(**e**). (**f**)–(**h**) AFM images of the Pt NPs fabricated with the In_2 nm_/Pt_2 nm_, In_30 nm_/Pt_30 nm_, and In_45 nm_/Pt_15 nm_ bilayers at 900 °C for 450 s. (**f-1**)–(**h-1**) Cross-sectional line profiles of Pt NPs in (**f**)–(**h**).

In this paper, taking it into another step, the modified solid state dewetting (MSSD) of Pt films by using In layer has been extended to fabricate very small (< 40 nm), medium (~350 nm) Pt NPs and large Pt networks (> 1000 nm) by utilizing several combinations of In/Pt bilayers on sapphire (0001) and their corresponding optical properties are thoroughly investigated along with the electro-magnetic field simulation. In addition, the underlying fabrication mechanism of Pt nanostructures by the MSSD is systematically described through the concurrent effects of inter-mixing of atoms, diffusion of alloy atoms and sublimation of In atoms in Figure 1a,b. The In/Pt bilayer configuration can promote the inter-mixing of In and Pt atoms, forming the In-Pt alloy and the In atoms can sublimate from the NP matrix, thus conveniently yielding nearly pure Pt NPs. With the In_2 nm_/Pt_2 nm_ bilayers, very small dense NPs are demonstrated. The In_45 nm_/Pt_15 nm_ bilayers annealed between 650 and 900 °C for 450 s demonstrate intermediate size of NPs with much improved interparticle spacings. Widely connected network-like nanostructures are demonstrated with the In_30 nm_/Pt_30 nm_ bilayers. The corresponding optical properties of Pt nanostructures are studied in terms of reflectance, transmittance and extinction, which exhibits the dynamic LSPR modes depending upon the morphology of Pt nanostructures. Specifically, the small Pt NPs exhibit the quadrupolar (QP) at UV region and dipolar (DP) resonance modes at VIS region while the large Pt NPs show the higher order (HO) and multipolar (MP) resonance modes respectively. The intermediate NPs demonstrate mixed transitional behaviors. The intensity and distribution of localized E-field largely depends upon the size and configuration of Pt NPs.

## 2. Materials and Methods

The fabrication of Pt nanostructures was carried out on 430 micron-thick transparent sapphire (0001). Initially, the sapphire wafers with an off-axis of ± 0.1° (iNexus Inc., Seongbuk-gu, Seoul, South Korea) were diced into small 6 × 6 mm^2^ pieces and cleaned via the degassing process in a pulsed laser deposition (PLD, DaDa TG Co. Ltd., Dalseong-gun, Daegu-si, South Korea) chamber at 600 °C for 30 min under 1 × 10^−4^ Torr. The surface morphology of degassed sapphire samples appeared atomically smoother as compared with the bare sapphire after the thermal removal of surface contaminants: i.e., various particulates, vapors and oxides as shown in Figure S1. In the next step, the indium (In) layer was deposited on a sample and then Pt film deposition was followed on top. Both In and Pt films were deposited at a growth rate of 0.05 nm/s with 3 mA ionization current under the vacuum of 1 × 10^−1^ Torr by means of sputtering in an ion-coater. In this experiment, three types of In/Pt bilayer series were prepared such as (a) In30 nm/Pt30 nm, (b) In2 nm/Pt2 nm and (c) In45 nm/Pt15 nm bilayer as shown in Figure S2a–c. Once ready, the annealing of as-deposited sample was performed in a PLD chamber at annealing temperature (Ta) between 500 and 900 °C under 1 × 10^−4^ torr with the linear control of temperature by 4 °C s^−1^. After reaching each target temperature, a constant dwelling duration of 450 s was given for all samples and the whole process was controlled by a computer recipe. After the completion of NP fabrication, the temperature was quenched down to an ambient temperature under vacuum.

The fabricated Pt nanostructures were imaged by an atomic force microscope (AFM, Park Systems Corp., Suwon, South Korea) in an ambient environment and analyzed with the XEI software (XE-70, Park Systems Corp., Suwon, South Korea). Also, the large-scale morphologies of Pt nanostructures were obtained by the scanning electron microscope (SEM, COXEM Co. Ltd., Yuseong-gu, Daejeon, South Korea) in vacuum. The elemental analysis of Pt nanostructures was performed with an energy-dispersive X-ray spectroscope (EDS, Noran System 7, Thermo Fisher Scientific, Waltham, MA, USA). The optical properties of Pt NPs such as reflectance and transmittance were characterized using an NOST system (Nostoptiks Co. Ltd., Seongnam-si, Gyeonggi-do, South Korea) at an ambient environment. The electric field distribution of Pt NPs on sapphire were simulated using FDTD solver (Lumerical Solutions, Vancouver, BC, Canada). The 3D surfaces of typical NPs were recreated by importing AFM images in an object space. The plane wave excitation source ranged from 250 to 1100 nm was engaged above each structure along the z-axis and the polarization of light was along x-axis and incident on the structure normally. The simulation boundaries were PML on z-axis and periodic on x- and y-axis. Two different observation planes were used to observe the local electric field in x-y and x-z planes. The refractive index for sapphire and Pt were taken from Palik’s model and fitted within 250 and 1100 nm [32].

## 3. Results and Discussion

Figure 1 presents the modified solid state dewetting (MSSD) in a sharp contrast to the conventional SSD of the pure Pt films by using a sacrificial In layer. The SSD is a self-assembly approach of transforming metastable thin films into arrays of nanostructures via the thermal annealing below the melting point of elements utilized as shown in Figure 1(a,a-1) [33,34]. Upon annealing, the metastable metallic thin films can undergo the SSD due to the diffusion of metallic adatoms [35] and the dewetting degree of metallic films can be controlled by the Ta, duration, film thickness as well as the diffusion kinetics of atoms and substrates [26]. Generally, the dewetting process can be primarily controlled by the Ta because it is largely related to the diffusion length and diffusivity of adatoms [33]. The diffusion of atoms and thus the evolution of surface morphology is directly related to the Ta as given by the diffusion coefficient $\left(D\right)=D_{0}\;\exp\left(-\frac{E_{a}}{\mathrm{kT}}\right)$., where the D_0_, E_a_, k and T are pre-exponential diffusivity, activation energy, Boltzmann constant and Ta respectively [36].

Meanwhile, however, the diffusivity of Pt adatoms is quite low even at high temperature as compared with the Au or Ag atoms [26,29,33] and thus the pure Pt films can only yield irregular or un-uniform Pt nanostructures by the conventional SSD. Indeed, the sacrificial In layer between the Pt film and substrate can enhance the dewetting to form improved configuration of Pt NPs with the fixed Pt layers thickness (10 nm) as well as with the various In/Pt thickness ratios in our previous works [30,31]. By the variation of In layer thickness under an identical growth condition and Pt thickness, the MSSD of In/Pt was found to alter the resulting Pt NPs: i.e., distinct and improved size and spacing. Furthermore, in this work, the MSSD approach is utilized to tune the areal density, size and configuration of Pt NPs widely by the systematic control of In and Pt layer thickness. This is due to the fact that the individual layer thickness of In and Pt directly affects the dewetting process and thus the evolution of NPs will be altered largely. The intermediate In layer can suffice the dewetting process due to its low surface energy (638 mJ/m^2^), high diffusivity and low melting point (156.6 °C) [37]. Upon annealing, the In and Pt adatoms can diffuse in both directions due to the inter-mixing at the In/Pt interface, which leads to an alloy phase of In and Pt atoms [38]. Due to the addition of highly diffusive In, the alloy can now have much higher diffusivity. Subsequently, the In atoms can sublimate from the NP matrix, even at a low temperature at ~ 350 °C and the rate of sublimation can exponentially increase with the increased temperature. As a result, the In atoms can extensively sublimate from the In-Pt alloy matrix at the usual growth temperatures and well-defined nearly pure Pt nanostructures can be obtained.

From the MSSD, the large enhancement on the configuration, size and density of Pt NPs can be achieved under the identical growth condition and Pt thickness. As an example, the comparative study on the surface morphology of Pt NPs fabricated at constant Ta of 900 °C and duration of 450 s with the pure films (2, 15 and 30 nm) and with the In/Pt bilayer (In_2 nm_/Pt_2 nm_, In_45 nm_/Pt_15 nm_ and In_30 nm_/Pt_30 nm_) films is presented in Figure 1c–e and Figure 1f–h respectively. As clearly observed by the AFM images, the resulting Pt NPs were largely improved by the MSSD under the identical growth conditions in terms of size, shape and spacing by the presence of In component. For instance, with the pure Pt film of 2 nm thickness, tiny and dense Pt NPs of < 3 nm height and < 20 nm diameter were obtained as shown in Figure 1(c,c-1). In contrast, the Pt NPs of average height ~ 10 nm, diameter ~ 40 nm and spacing ~ 50 nm were observed with the In_2 nm_/Pt_2 nm_ bilayer as displayed in Figure 1(f,f-1). Similarly, the 15 and 30 nm thick Pt film also showed great morphology enhancement with the sacrificial In layers. The detailed analysis on the improved dewetting of Pt NPs by using the sacrificial In layer is presented in the following sections by varying the Ta and thickness ratio of In/Pt bilayers.

**Figure 2:**
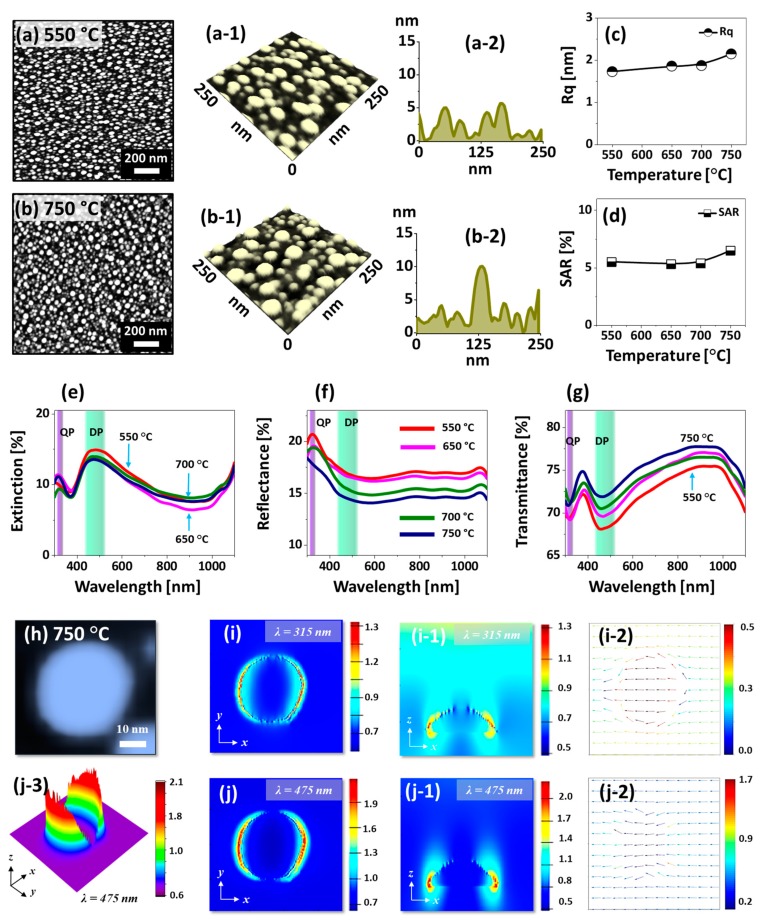
Small and dense Pt NPs on sapphire (0001) with the In_2 nm_/Pt_2 nm_ bilayers by annealing at 550 and 750 °C for 450 s. Other samples in the series are provided in the supplementary materials. (**a**)–(**b**) AFM top-views (1 × 1 µm^2^). (**a-1**)–(**b-1**) Enlarged AFM side-views (250 × 250 nm^2^). (**a-2**)–(**b-2**) Cross-sectional line-profiles. (**c**)–(**d**) Summary plots of RMS roughness (Rq) and surface area ratio (SAR). (**e**)–(**g**) Extinction, reflectance and transmittance spectra of Pt NPs. The color band in UV and VIS region depict the quadrupolar (QP) and dipolar (DP) resonance modes in (**e**)–(**g**). (**h**) AFM images of typical Pt NPs selected for the finite difference time domain (FDTD) simulations. (**i**)–(**j**) Local E-field distribution on Pt NPs in xy-plane. (**i-1**)–(**j-1**) E-field distribution in xz-plane. (**i-2**)–(**j-2**) E-field vector plots in xy-plane. (**j-3**) 3D-view of the local e-field distribution.

Figure 2 shows the morphological and optical analysis of densely packed small Pt NPs fabricated with the In_2 nm_/Pt_2 nm_ bilayer at 550 and 750 °C. This set was designed to analyze the effect of thin In/Pt bilayers on the evolution of Pt NPs at various Ta and other samples in the series are provided in Figure S3. Upon annealing of In/Pt bilayer, the high diffusivity In atoms can diffuse into the Pt layer and as a result the In and Pt atoms can be inter-mixed. As discussed, this can form the In-Pt alloy and then the In-Pt alloy atoms can diffuse together, creating atomic vacancies and agglomerates [33,34]. With the very thin In/Pt bilayers, the inter-mixing of metallic atoms as well as the overall dewetting can be significantly accelerated [39]. Thus, the highly dense, small and uniform Pt NPs can be resulted with the thin In_2 nm_/Pt_2 nm_ bilayers. For instance, at 550 °C, the densely packed round Pt NPs of average height ~ 6 nm and diameter ~ 40 nm were formed as clearly evidenced by the AFM top-view, side-view and cross-sectional line-profile in Figure 2(a,a-2). When the Ta was increased to 750 °C, the size of the Pt NPs was mildly increased whereas the density was slightly reduced, which can be described based on the coalescence of Pt NPs along with the increased diffusion of atoms at higher temperature. The Pt NPs obtained in this sets were much larger, uniform and isolated than those were demonstrated with the pure Pt film under the identical growth condition in the previous study [29]. Furthermore, as the Pt NPs could attain stable configurations even at low temperature, the evolution was not significantly occurred along with the Ta: i.e., the saturated growth of Pt NPs was observed in general. Therefore, the average height and diameter of the Pt NPs were ~ 10 and 60 nm respectively as clearly shown in Figure 2(b-1,b-2). Furthermore, the surface morphology of corresponding Pt NPs was further analyzed by the plots of RMS roughness (Rq) and surface area ratio (SAR) as shown in Figure 2c,d. The Rq and SAR were generally mildly increased between 550 and 750 °C due to the slightly increased height and surface area of Pt NPs. The elemental analysis of the samples by EDS spectra measurement is presented in Figure S4, which clearly showed the constant Pt Mα1 peak while the In peak was not observed. This indicates that the amount of Pt was constant for all samples and In was completely sublimated in the Ta range.

**Figure 3:**
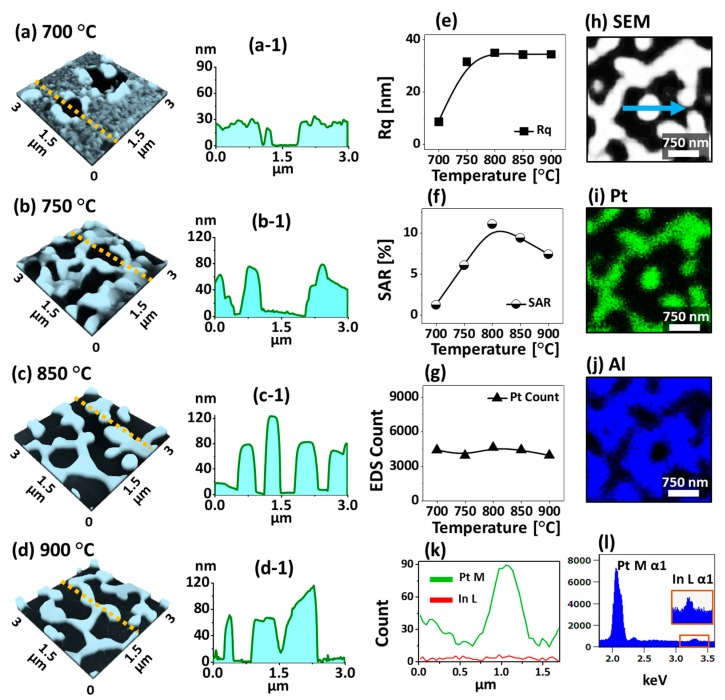
Evolution of various morphologies of Pt nanostructures with the In_30 nm_/Pt_30 nm_ bilayers by annealing between 700 and 900 °C for 450 s. (**a**)–(**d**) AFM side-views (3 × 3 µm^2^). (**a-1**)–(**d-1**) Cross-sectional line-profiles from (**a**)–(**d**). (**e**)–(**g**) Summary plots of Rq, SAR and energy dispersive x-ray spectroscopy (EDS) Pt count of the Pt nanostructures on sapphire. (**h**) SEM image (3 × 3 µm^2^) of sample annealed at 900 °C for 450 s. (**i**)–(**j**) Corresponding Pt and Al phase maps. (**k**) Elemental line-profile of Pt NP marked with an arrow in (**h**). (**l**) EDS spectrum of Pt nanostructures.

The corresponding optical properties of densely packed small Pt NPs fabricated with the In_2 nm_/Pt_2 nm_ bilayer are presented in Figure 2e–g. The reflectance and transmittance spectra were experimentally measured whereas the extinction spectra were extracted by using the relation: extinction [%] = 100% − reflectance [%] − transmittance [%]. The optical spectra demonstrated various shape such as peaks and dips at specific wavelength between 300–1100 nm depending on the surface morphology of Pt nanostructures. In the extinction spectra, a weak absorption peak at UV (~ 320 nm) and intense absorption peak at VIS region (~ 480 nm) were observed as shown in Figure 2e. With the very small sized Pt nanostructures, the peak at UV and VIS region can be caused by the quadrupolar (QP) and dipolar (DP) resonance mode respectively [40]. Since the DP resonance is pronounced with the small size Pt NPs, the absorption peak at VIS region was much intense than the UV peak. The corresponding E-field distribution at QP and DP resonance were demonstrated with the typical Pt NP in Figure 2h–j. In general, the E-field was confined on both side of NPs forming strong lobes as seen in the 3D map in Figure 2(j-3). From the E-field profile at QP and DP, it is clearly observed that the visible region at 475 nm showed a stronger field enhancement as compared to the UV at 315 nm, which agrees well with the intensity of extinction peaks. The E-field vector plot of QP and DP showed directions along the axis while few were directed away as shown in Figure 2(i-2,j-2). At longer wavelength in NIR region, the extinction dip was observed in Figure 2e, which can be caused by the high transmittance or low plasmonic resonance absorption. The extinction spectra were similar for all the samples due to the similar size and surface morphology of Pt NPs along with the early saturation of growth by the diffusion enhancement as discussed. The corresponding reflectance spectra of Pt NPs in Figure 2f demonstrated a narrow peak at UV region and gradual downward slope towards the VIS region and flat spectral shape at NIR region. From the reflectance, the absorption dip was not obvious, which can be due to the enhanced backscattering of photons with the strong dipolar resonance of small Pt NPs [41,42,43]. From the transmittance spectra in Figure 2g, a narrow dip at ~ 315 nm and a broad dip at ~ 460 nm was observed based on the QP and DP resonance mode respectively as discussed [41]. In terms of the spectral shape, all the transmittance plots were quite similar within the Ta range with the variation in average value again due to the similar morphology of NPs. Depending upon the mild evolution of Pt NPs, the average reflectance was decreased whereas the average transmittance was increased as a function of the Ta.

Figure 3 shows the evolution of Pt nanostructures with the In _30 nm_/Pt _30 nm_ bilayers along with the Ta between 700 and 900 °C for 450 s. The surface morphologies of Pt nanostructures such as the void, connected and isolated nanostructures at a series of Ta are documented by the AFM side-views and cross-sectional line-profiles. The large-scale SEM images and low temperature samples are presented in supplementary Figure S5. In contrast to the previous set, the dewetting degree was largely suppressed at low annealing temperature such that below 700 °C only minor surface evolution was observed. This could be likely due to the insufficient diffusion of atoms for thick In/Pt bilayer. As shown in Figure 3(a,a-1), the annealing of In _30 nm_/Pt _30 nm_ bilayer at 700 °C resulted in the formation of large voids with the depth of ~ 26 nm. The voids on thick In/Pt bilayer can be formed by the coalescence of atomic vacancies, which gradually become larger due to the enhanced diffusion and agglomeration of atoms at increasing temperature. Therefore, when the Ta was increased to 750 °C, the connected Pt nanostructures were formed from the partially voided film as shown in Figure 3(b,b-1). As the Ta was further increased between 800 ~ 900 °C, the nanostructures were further developed into the network-like pattern. Meanwhile, some isolated Pt NPs were resulted based on the Rayleigh instability of large nanoclusters [44].

The average height of Pt nanostructures and the exposed surface area of sapphire substrate were significantly increased due to the compact agglomeration of atoms as clearly demonstrated in Figure 3c,d and Figure 3(c-1,d-1). By comparing with the 30 nm thick pure Pt film dewetting, these NPs with the MSSD were found to be more connected, which could be due to the coalescence growth of Pt nanoclusters at favorable diffusion conditions. The variation in Pt nanostructures height and surface area at different Ta was further studied in terms of Rq and SAR as shown in Figure 3e,f. When the Ta was increased between 700 and 800 °C, both the Rq and SAR were gradually increased to 34.33 nm and 9.41% respectively along with the increased vertical height and surface area exerted by the Pt nanostructures. However, above 850 °C, the Rq remained similar while the SAR was decreased to 7.42% due to the reduction of surface area possessed by the Pt nanostructures. Furthermore, the corresponding EDS counts plot depicts almost identical counts of Pt between 700 and 900 °C, indicating the equal amount of Pt deposition although the morphology is dissimilar as clearly shown by the plot in Figure 3g. The elemental analysis of Pt nanostructures fabricated with the In_30 nm_/Pt_30 nm_ bilayer annealed at 900 °C is investigated in terms of SEM image, EDS maps, spectral line-profile and spectra as shown in Figure 3h–l. The SEM image (h), Pt Map (g) and Al map (h) are perfectly matching with each other, which indicates the nanostructures are consisted of Pt atoms. Furthermore, the spectral line-profile of Pt nanostructures at specific region is analyzed, which shows the high counts of Pt and minor count of In as shown in Figure 3k. Similarly, the EDS spectra of Pt nanostructures evidenced the much higher Pt counts and lower In count (at around 3.286 keV) as shown in Figure 3l. The subtle presence of In atoms even at high Ta can be likely due to the formation of In-Pt intermetallic phases based on the In-Pt equilibrium In-Pt phase diagram [38]. Another series of sample with constant Pt but reduced In thickness, I.e. In_10 nm_/Pt_30 nm_ was also studied, which demonstrated much reduced dewetting extent as shown in Figures S9–S13. This clearly indicated that the dewetting degree of Pt NPs can be altered by varying the thickness In layer.

**Figure 4:**
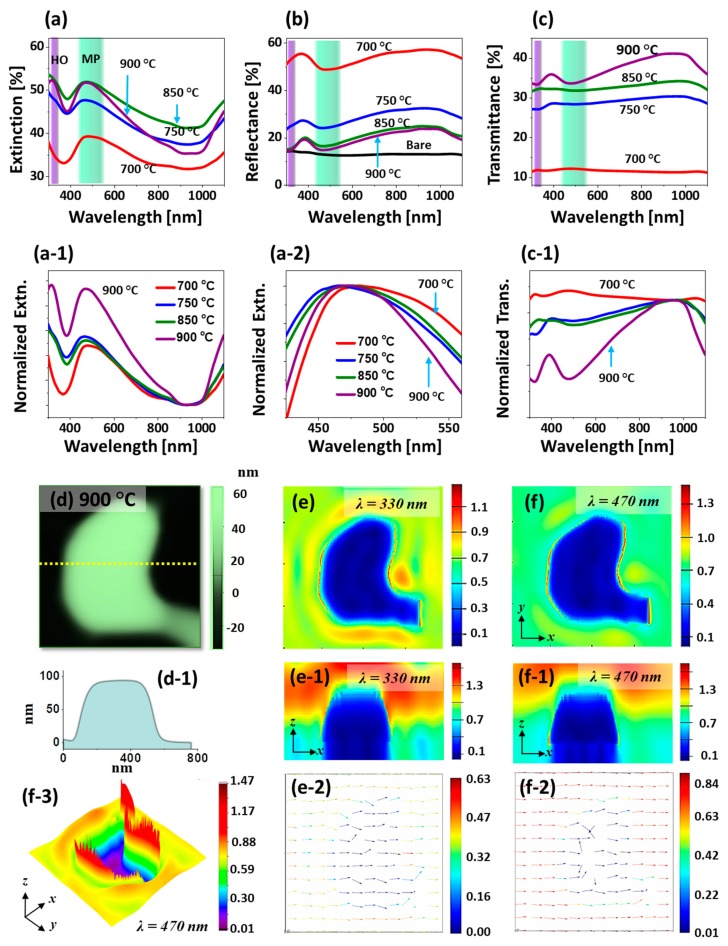
LSPR properties of Pt nanostructures fabricated with the In_30 nm_/Pt_30 nm_ bilayers at various annealing temperature. (**a**)–(**a-2**) Extinction, normalized extinction and magnified extinction spectra. (**b**) Reflectance spectra. (**c**)–(**c-1**) Transmittance and normalized transmittance spectra. The color bands at the UV and VIS regions in (**a**)–(**c**) denote the higher order (HO) and multi-polar (MP) resonance modes respectively. (**d**) AFM images of typical Pt NPs. (**d-1**) Cross-sectional line-profiles. (**e**)–(**f**) E-field profiles of NPs in xy-plane at different wavelengths. (**e-1**)–(**f-1**) E-field profiles in xz-plane. (**e-2**)–(**f-2**) E-field vector plots in xy-plane. (**f-3**) 3D-view of the e-field distribution.

Figure 4 shows the optical properties of corresponding large Pt nanostructures in terms of optical spectra and FDTD simulation. As compared with the previous set, the Pt NPs are much larger and thus distinctive spectral shape and plasmon resonance are observed as shown by the extinction, reflectance and transmittance spectra in Figure 4a–c. For instance, the extinction spectra in Figure 4a revealed a narrow absorption peak in the UV region and a broader absorption peak in the VIS region. When the Pt nanostructures are very large, the multi-polar (MP) resonance at the VIS region and higher order (HO) resonance at a shorter wavelength range, i.e., at the UV region, can be induced with the interaction of incident light [40,41]. In specific, the MP resonance can be generated by the superimposition of the various resonance modes such as quadrupolar, dipolar and other resonance modes. Therefore, the most significant absorption peaks in the VIS wavelength can be contributed by the MP resonance modes of large Pt nanoclusters. In addition, the local E-field enhancement at the resonance wavelength were simulated in the FDTD software. The typical irregular Pt NP fabricated at 750 °C had the irregular configuration as shown in Figure 4d, which shows a strong e-filed at multiple boundary regions. The E-field corresponding to the HO and MP resonance modes, i.e., at 330 and 465 nm respectively, are displayed in Figure 4(e,e-1) and Figure 4(f,f-1). The intensity of E-field at 465 nm was slightly stronger than at 330 nm likely due to the stronger resonance in the visible region. From the 3D view of E-field profile in Figure 4(f-3), the region where the localized E-field was strong can be seen clearly. The E-field vector at 330 and 465 nm were mostly along the axis while few of them were directed away as shown in Figure 4(e-2,f-2). The intensity of resonance peak was gradually increased with the increased Ta as shown by the normalized extinction spectra in Figure 4(a-1), which signifies the enhanced absorption in the UV and VIS wavelength along with the morphological transition from widely connected to isolated Pt nanostructures.

Furthermore, as shown by enlarged spectra in Figure 4(a-2), the width of MP resonance peak was gradually narrowed with the increased Ta because of the reduced size distribution of Pt nanostructures by the transition from the layered to the isolated morphology [41]. On the other hand, the extinction spectra also exhibited the dip at NIR region as shown in Figure 4(a,a-1), which can be correlated to the pronounced reflectance and/or transmittance shoulders. Furthermore, the reflectance spectra in Figure 4b demonstrated the formation of absorption dip in the UV and VIS regions as a consequence of HO and MP resonance mode of larger Pt nanostructures as discussed [40,41]. Generally, within the Ta range between 700 and 900 °C, the spectral shape of reflectance was somewhat similar, and the dip intensity was slightly decreased. This could be likely due to the scattering dependence on the size evolution of Pt NPs. On the other hand, the transmittance spectra showed the sharp distinction in the spectral evolution as shown in Figure 4(c,c-2). Like the absorption in extinction and reflectance spectra, the absorption dip can be expected in the VIS region in the transmittance spectra. However, the flat shoulder was observed at visible wavelength for the connected Pt nanoclusters at low temperature as shown in Figure 4(c-1), which can be likely due to the pronounced forward scattering of photons with large nanostructures. With the formation of network-like and isolated Pt nanostructures at high Ta of 900 °C, the absorption dip became obvious based on the MP resonance mode of large Pt nanostructures [30]. This could be due to the pronounced dipolar resonance in the visible region with much isolated Pt NPs although other resonance modes can also contribute to the LSPR effect. As shown by the plot in Figure 4(c-1), the enhanced average transmittance of light was observed as a function of Ta due to the reduced surface coverage of Pt nanoclusters.

**Figure 5:**
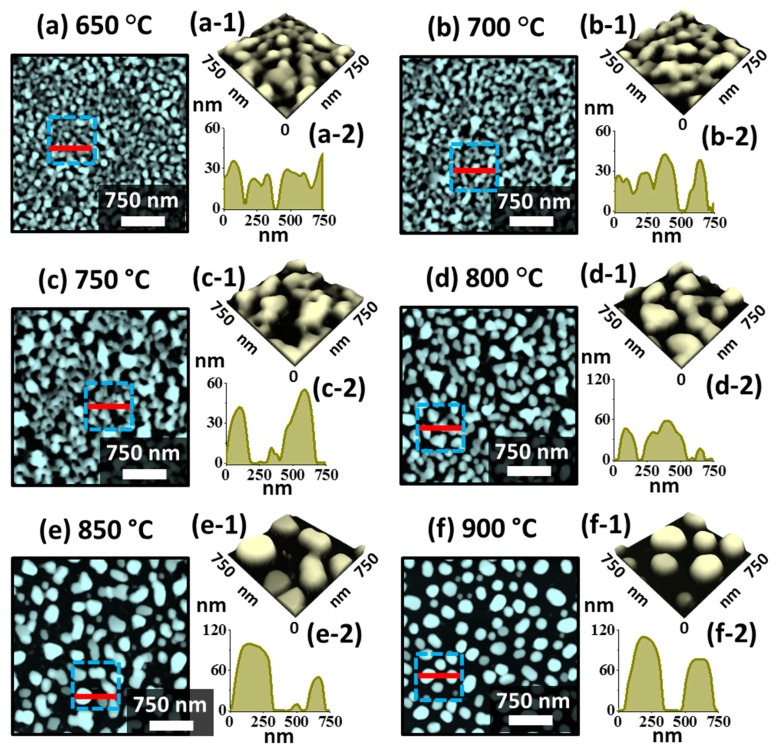
Evolution of medium size and isolated Pt NPs with the In_45 nm_/Pt_15 nm_ bilayers annealed between 650 and 900 °C for 450 s. (**a**)–(**f**) AFM top-views of 3 × 3 µm^2^. (**a-1**)–(**f-1**) Magnified side-views (750 × 750 nm^2^) of corresponding Pt nanostructures (boxed regions in the AFM top-views). (**a-2**)–(**f-2**) Cross-sectional line-profiles of the regions marked with the red lines in (**a**)–(**f**).

Figure 5 shows the evolution of medium sized Pt NPs on sapphire (0001) by annealing In_45 nm_/Pt_15 nm_ bilayers between 650 and 900 °C for 450 s. In contrast to the previous two sets, the Pt NPs in this set show the intermediate stage in terms of size, configurations and LSPR response. In general, the connected, elongated and isolated Pt nanostructures were formed along with the increasing Ta.

Although the total thickness was fixed at 60 nm, the same as the previous set (In_30 nm_/Pt_30 nm_), the In layer thickness was increased to 45 nm and the Pt layer thickness was reduced to 15 nm. While the total thickness of film is a determining factor of diffusion in the MSSD, the temperature dependent dewetting of bilayer film can also be strongly affected by the individual thickness of constituent elements. With the increased thickness of In component the overall dewetting of In/Pt bilayer was significantly enhanced in this case, which can be correlated to the high diffusivity of In atoms [39,45]. As mentioned, this can be due to the enhanced inter-diffusion and alloying between In-Pt at the In/Pt interface, which eventually enhanced the global diffusion of the overall system. Further, the dewetting process can be more dynamic at increased temperature due to the large sublimation loss of In atoms. Initially, at the Ta of 650 °C, the connected Pt nanostructures and voids were observed, and the height of typical Pt nanostructures was between 20 ~ 40 nm as shown in Figure 5(a,a-2). At increased temperature, the voids grew larger by merging the nearby ones and ultimately the edges of the void can fragment and lead to the formation of connected nanostructures. This can be observed up to 750 °C as shown in Figure 5b,c as the initial Pt top layer was quite thick. Meanwhile, the size of Pt nanostructures was increased significantly exposing large portion of the substrate area. The Pt nanostructures were still slightly elongated and irregular because of the insufficient thermal energy for the large mass of Pt. Consequently, as shown in Figure 5d–f, the Pt NPs were gradually transformed into the isolated configuration between 800 and 850 °C based on the Rayleigh instability [39]. Finally, the semi-spherical Pt NPs were obtained at 900 °C as shown in Figure 5f, owing to the preferential isotropic surface energy distribution of NPs to become stable [45]. The average height and diameter of Pt NPs were increased up to ~ 110 and 350 nm respectively. As compared to the previous study on pure Pt film of similar thickness, the Pt NPs showed great enhancement in terms of uniformity, size, spacing and configurations [36].

**Figure 6:**
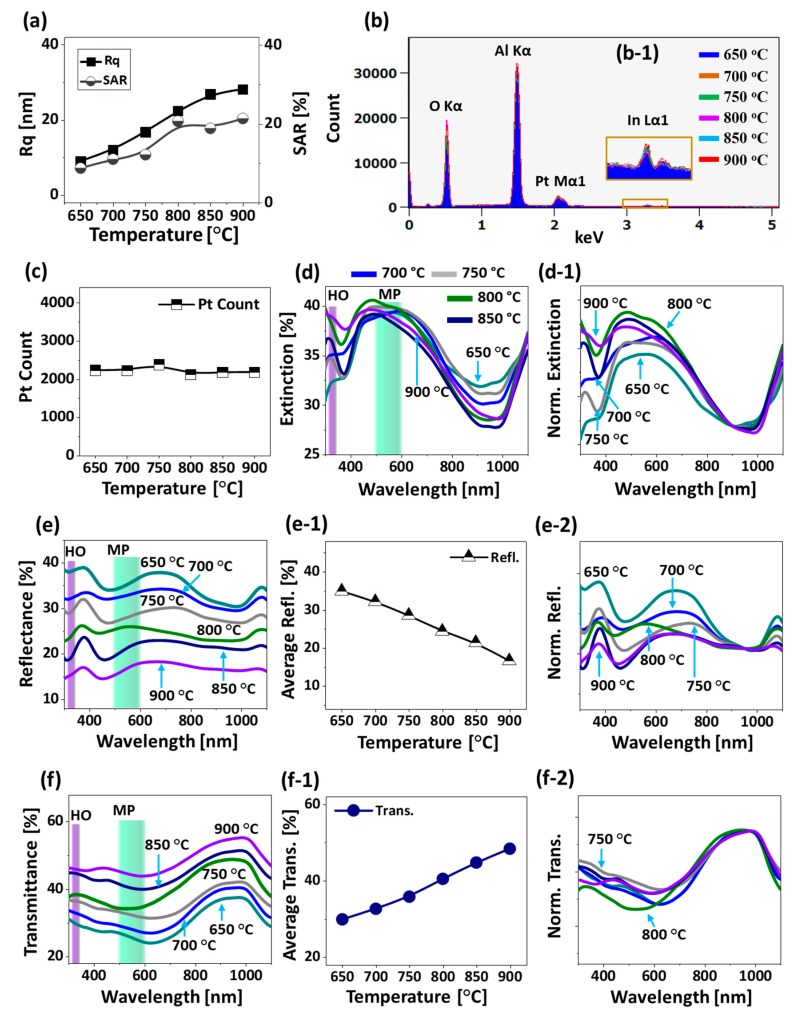
Morphological, elemental and optical summaries of Pt NPs with the In_45 nm_/Pt_15 nm_ bilayers. (**a**) Summary plots of Rq and SAR. (**b**) EDS spectra of Pt NPs. (**b-1**) (Inset) Enlarged In peak at ~3.2 keV. (**c**) Pt count at different temperatures. (**d**)–(**d-1**) Extinction and normalized spectra. (**e**)–(**e-2**) Reflectance, average reflectance plot and normalized spectra. (**f**)–(**f-2**) Transmittance spectra, average transmittance plot and normalized spectra.

Additional analysis of the morphological evolution of Pt NPs is performed in terms of Rq and SAR as presented in Figure 6a and both the Rq and SAR were gradually increased along with the Ta as the average height and surface area of Pt NPs were increased. The elemental analysis of Pt nanostructures is performed by the EDS spectra measurement as shown in Figure 6b,c. The EDS spectra clearly showed the presence of O Kα, Al Kα, Pt Mα1 and In Lα1 peaks and the intensity of Pt Mα1 was very high as compared with the In Lα1 because of the extensive sublimation of In atoms. Figure 6(b-1) shows a minor In Lα1 peak at around 3.286 keV from the boxed region in Figure 6b, which can be due to the In-Pt intermetallic phases [38]. From the summary plot of Pt counts in Figure 6c, it was found that the amount of Pt was same in all the samples regardless of the distinct Pt nanostructures throughout the Ta range. As compared with the previous sets, distinctive morphology of Pt NPs was realized in this set such as the intermediate degree of NPs size, spacing and uniformity from the first and second sets while much improved morphology was observed as compared to the pure Pt dewetting [36]. The optical properties of corresponding Pt nanostructures fabricated with the In_45 nm_/Pt_15 nm_ bilayer are presented in Figure 6d–f. The medium sized Pt NPs clearly demonstrated distinct optical properties in comparison with the small and large Pt NPs in the previous two sets. In specific, the extinction spectra showed a weak absorption peak in the UV region and a broad absorption peak in the VIS region as shown in Figure 6d and the NPs demonstrated the intermediate behavior. Since the average size of the Pt NPs was greater than 300 nm, the absorption peaks at the UV and VIS region can be associated with the HO and MP resonance modes respectively [41].

**Figure 7:**
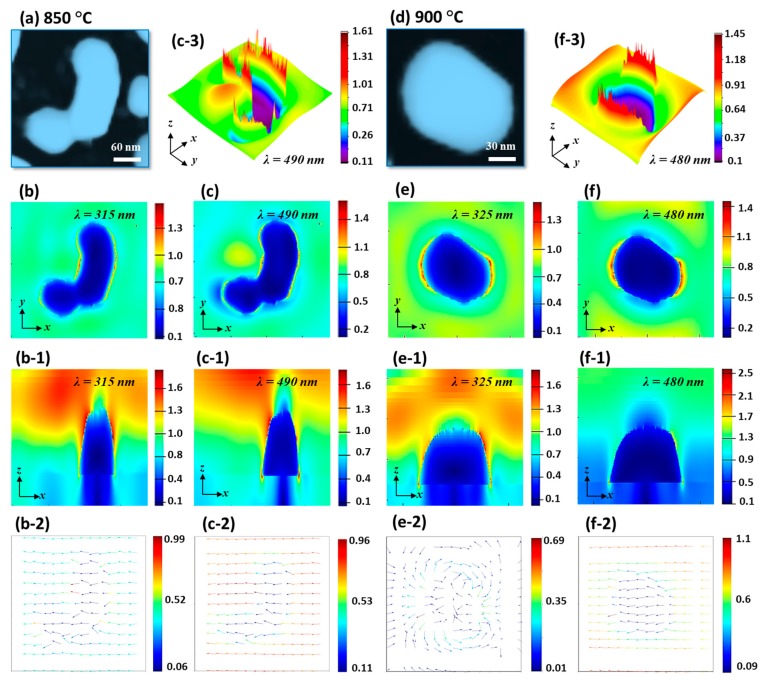
FDTD analysis of the medium size Pt NPs on sapphire fabricated with the In_45 nm_/Pt_15 nm_ bilayers. (**a**) and (**d**) AFM image of the Pt NPs at 850 and 900 °C. (**b**)–(**c**) and (**e**)–(**f**) E-field profiles of Pt NPs in xy-plane. (**b-1**)–(**c-1**) and (**e-1**)–(**f-1**) E-field profiles in xz-plane. (**b-2**)–(**c-2**) and (**e-2**)–(**f-2**) e-field vector plots. (**c-3**) and (**f-3**) 3D view of the e-field profiles at specific wavelength.

The E-field enhancement for typical irregular and regular NPs were determined by the FDTD simulation in Figure 7. In the case of irregular NP, the E-field at HO and MP are shown in Figure 7b,c among which the MP was found to be stronger. Generally, the E-field was stronger at the boundary of NPs at both resonance wavelengths. The E-field vectors were mostly along the axis while few were directed away for the HO and MP resonances. The extinction spectra further showed that the VIS absorption band was stronger, and boarder as compared with the previous two sets, which can be attributed to the superimposition of various resonance modes. Indeed, the absorption peak was the widest in this set out of the three. The intensity of MP resonance peak in VIS region was gradually enhanced along with the increased Ta as shown in Figure 6(d-1), indicating the increased absorption with the formation of isolated and larger Pt NPs. From the simulation of E-field of isolated and regular Pt NPs, the E-field enhancement was found to be much confined at the boundary of NP as displayed in Figure 7d–f. The e-filed vector was also largely altered with the rounder NPs as the HO showed random direction along the NP geometry while the MP resonance showed most of the vector along the axis as shown in Figure 7(e-2,f-2).

Furthermore, the reflectance spectra in Figure 6e demonstrated minor absorption dips at UV region corresponding to the HO resonance mode. However, the VIS region of reflectance spectra exhibited a dip commonly at ~ 480 and peak at ~ 700 nm for the low temperature samples and a broad shoulder for the high temperature samples. Since the MP resonance mode in the VIS region can be contributed by the dipolar, quadrupolar and other resonance modes, the strongest can influence the overall resonance mode based on the size of Pt NPs [46]. In order to investigate the effect of Pt nanostructures size on the reflectance spectra in detail, the original spectra were normalized as shown in Figure 6(e-2). In specific, the VIS peak showed a decreasing trend with the size increment of NPs and this can be since the low temperature samples had smaller NPs, which can give dipolar resonance behavior. This can yield the enhanced backscattering and thus the intense peak at ~ 700 nm can be formed. As the large and isolated NPs started to develop, the quadrupolar resonance can become more significant and thus the backscattering can be gradually reduced. Likewise, the visible peak and shoulder in reflectance can gradually attenuate along with the Ta. In terms of the transmittance spectra, it generally showed the formation of broad dip in the VIS region and strong peak in the NIR as seen in Figure 6(f,f-2). The absorption dip in the transmittance spectra can also be affected by the forward scattering of medium sized Pt NPs, which can cause the distortion in the shape of absorption dip [23,47]. From the transmittance spectra, the absorption enhancement in the UV-Vis region was clear with the formation of broad dip. Adding the reflectance and transmittance spectra analysis together, the broad extinction peaks can be due to the intermediate behavior of medium size NPs, namely the medium size NPs demonstrated the enhanced backward scattering at ~ 700 nm and the transmittance behaver was quite distinct from the previous two sets. Last, in terms of average reflectance and transmittance, they showed the opposite trend due to the gradual surface coverage change as before: i.e., gradually decrease reflectance along with the increased transmittance.

## 4. Conclusions

In summary, the improved morphology and LSPR properties of Pt nanostructures of very small (<40 nm), medium (~350 nm) Pt NPs and large Pt networks (>1000 nm) were successfully demonstrated through the modified SSD (MSSD) of In/Pt bilayers. The sacrificial In layer showed great improvement for the tuning configuration and uniformity of low diffusivity metallic elements such as Pt and the capability of wide-range tunability was demonstrated for various size Pt NPs. In terms of the LSPR behavior, generally improved LSPR response was demonstrated by virtue of the fabrication of well-structured, isolated and uniform Pt NPs. Depending on the size of Pt NPs, various plasmonic resonance modes were observed and the major extinction peaks were generally observed in the VIS region, which was further described by the FDTD simulation.

The In atoms can inter-mix with the Pt atoms and can form the In-Pt alloy owing to the low surface energy and high diffusivity. Well-developed Pt nanostructures were formed through the enhanced dewetting and subsequent sublimation of In atoms. Depending upon the annealing temperature and initial bilayer thickness, the resulting morphology of Pt nanostructures was significantly transformed within the range where the pure Pt films showed minor surface evolution. The dewetting enhancement was further exploited by using various In layer thickness and the results were contrasted with the previous literatures.

## Supplementary Materials

The Supplementary Materials are available online at https://www.mdpi.com/2079-4991/9/6/831/s1, the Supplementary Materials includes the bare substrate and various bilayer characterizations and additional morphological, elemental and optical characterizations of samples, including the AFM, SEM images, EDS spectra, FDTD simulations and optical spectra.
