# Supplementary material for: Fabrication of Various Plasmonic Pt Nanostructures via Indium Assisted Solid-State Dewetting: From Small Nanoparticles to Widely Connected Networks
Source: Nanomaterials (Basel). 2019 May 31;9(6):831. doi: 10.3390/nano9060831 (PMC6631651; doi:10.3390/nano9060831)
Supplement: Supplementary file 1 [file nanomaterials-09-00831-s001.pdf]

Supplementary Materials

Fabrication of Various Plasmonic Pt Nanostructures via Indium Assisted Solid-State Dewetting: From Small Nanoparticles to Widely Connected Networks

Sanchaya Pandit [1], Mao Sui [2,*], Sundar Kunwar [1], Puran Pandey [1], Sandesh Pant [1] and Jihoon Lee [1,*]

[1] Department of Electronic Engineering, College of Electronics and Information, Kwangwoon University, Nowon-gu Seoul 01897, South Korea; sanchaya7@gmail.com (S.P.); kunwarankees23@gmail.com (S.K.); ppcpurans@gmail.com (P.P.); sandeshpant2@gmail.com (S.P.)

[2] Institute of Hybrid Materials, College of Materials Science and Engineering, Qingdao University, Qingdao 266071, China

* Correspondence: maosui001@qdu.edu.cn (M.S.); jihoonlee@kw.ac.kr (J.L.)

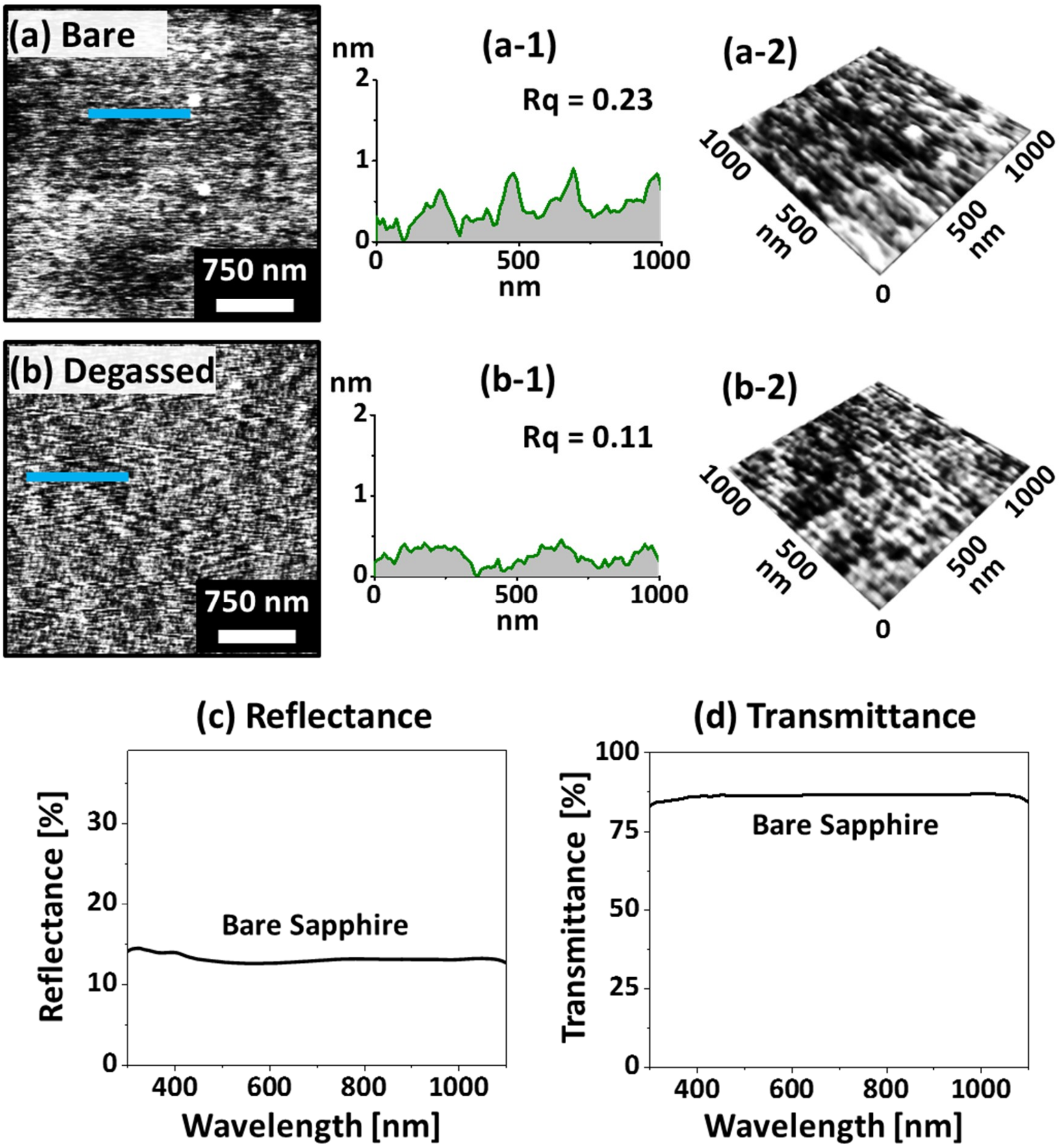

Figure S1: Surface morphology and optical properties of bare sapphire (0001). (a) – (b) AFM top-views (3 × 3 μm^2) of bare and degassed sapphire samples. (a-1) – (b-1) Cross-sectional line-profiles. (a-2) – (b-2) Magnified AFM side-views (1 × 1 μm^2). (c) – (d) Reflectance and transmittance spectra of bare sapphire.

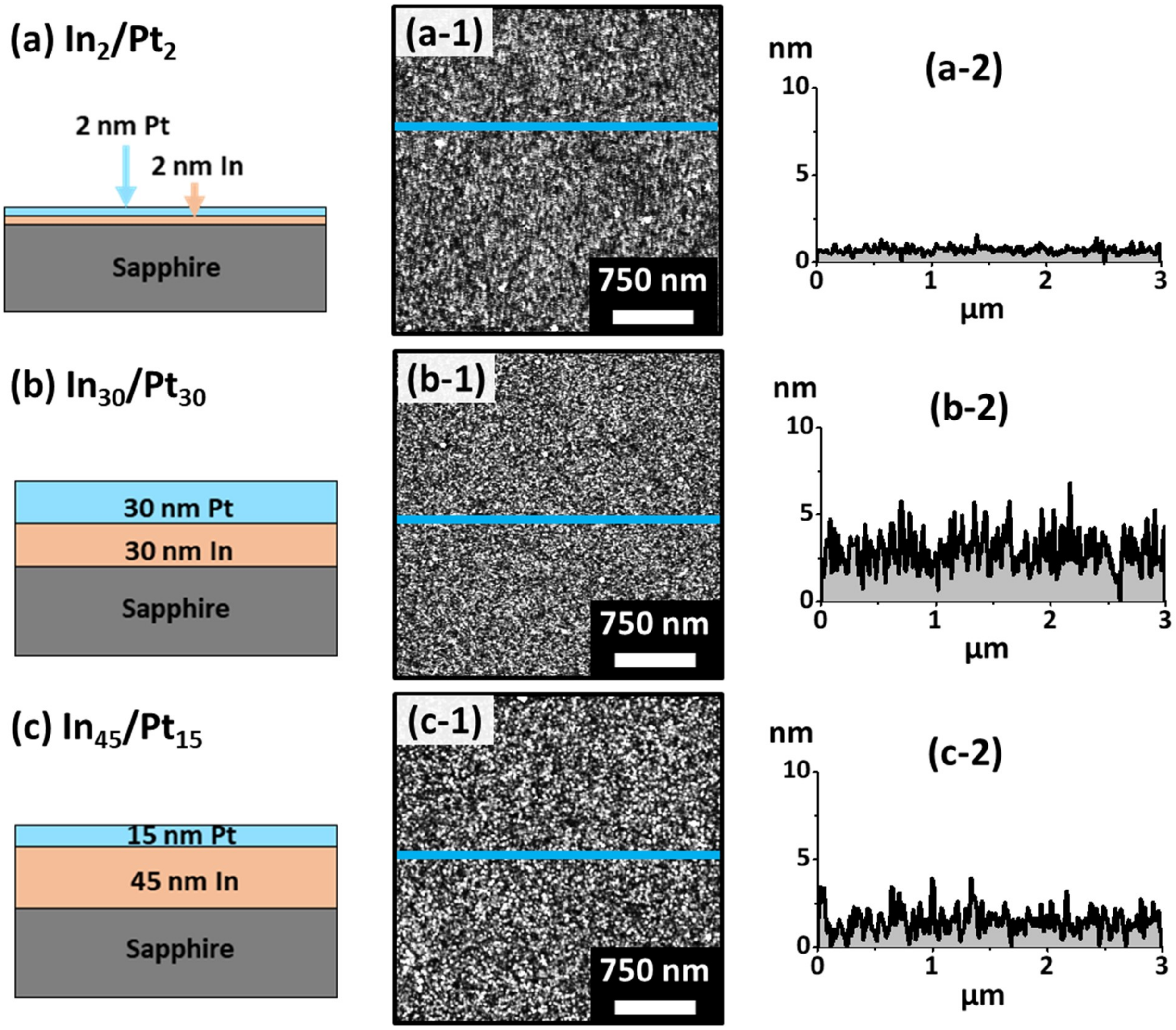

Figure S2: (a) – (c) Schematic of as-deposited In / Pt bilayers on sapphire (0001). (a-1) – (c-1) Corresponding AFM top-views (3 × 3 μm^2) after the deposition of $In_{30\ nm}$ / $Pt_{30\ nm}$, $In_{10\ nm}$ / $Pt_{30\ nm}$, $In_{2\ nm}$ / $Pt_{2\ nm}$ and $In_{45\ nm}$ / $Pt_{15\ nm}$ bilayers respectively. The $In_{30\ nm}$ / $Pt_{30\ nm}$ indicates that 30 nm of indium was first deposited and then 30 nm of platinum was added atop subsequently. (a-2) – (d-2) Corresponding cross-sectional line-profiles.

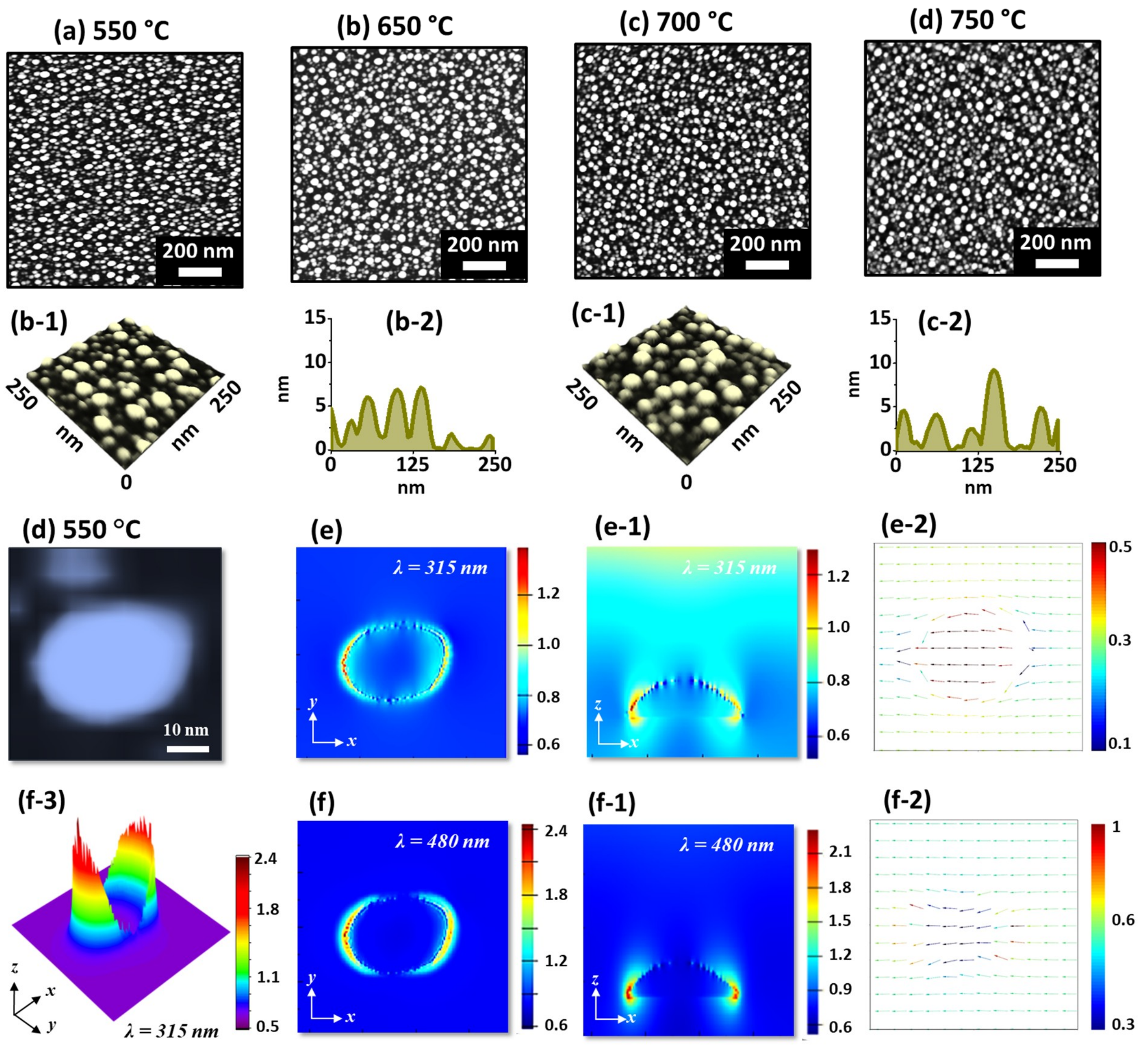

Figure S3: Small dense Pt nanoparticles (NPs) on sapphire (0001) fabricated with the $In_{2\,nm}$ / $Pt_{2\,nm}$ bilayers by annealing between 550 and 750 °C for 450 s. (a) – (d) AFM top-views (1 × 1 μm²). (b-1) – (c-1) Enlarged AFM side-views (250 × 250 nm²). (b-2) – (c-2) Cross-sectional line-profiles. (d) AFM images of typical Pt NPs selected for the FDTD simulations. (e) – (f) Local E-field distribution on Pt NPs in xy-plane. (e-1) – (f-1) E-field distribution in xz-plane. (e-2) – (f-2) E-field vector plots in xy-plane. (f-3) 3D-view of the local e-field distribution.

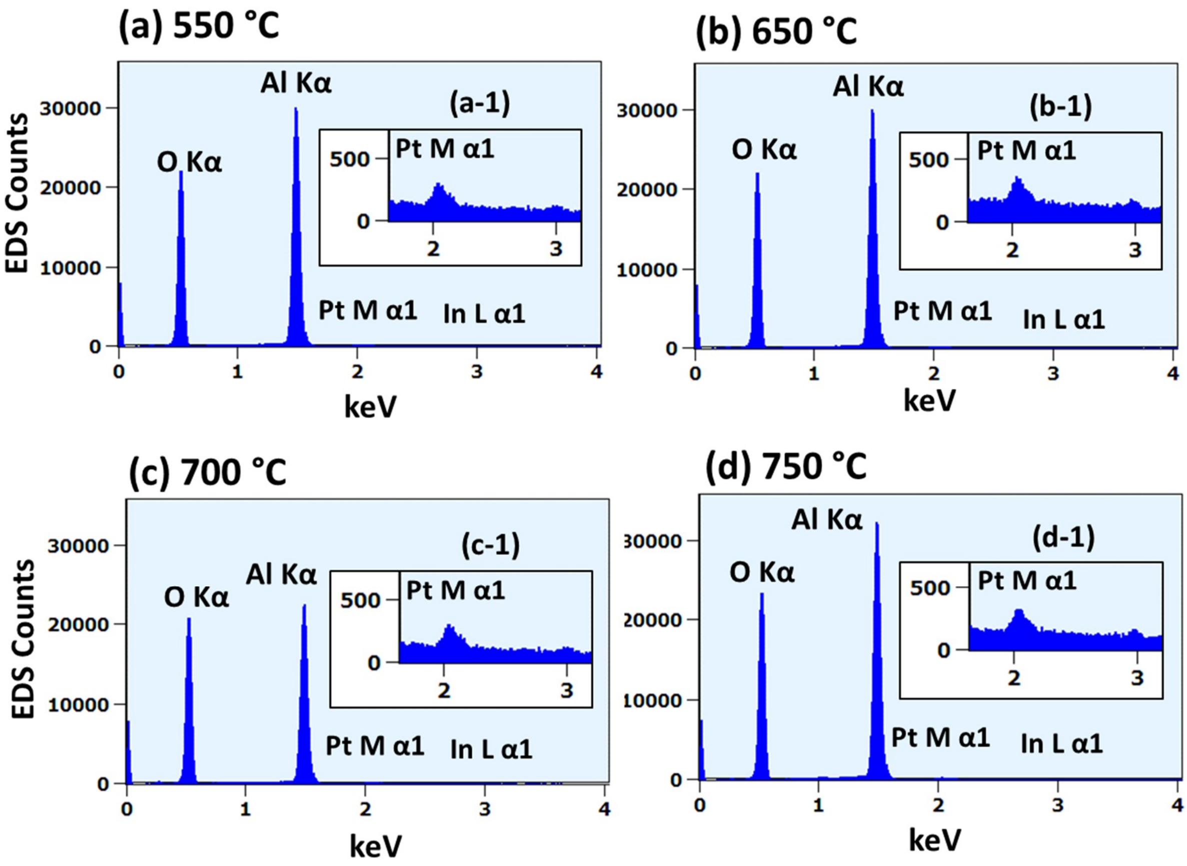

Figure S4: EDS spectra of densely packed Pt NPs on sapphire fabricated at various temperature between 550 and 750 °C by using the thin $In_{2\,nm}$ / $Pt_{2\,nm}$ bilayers.

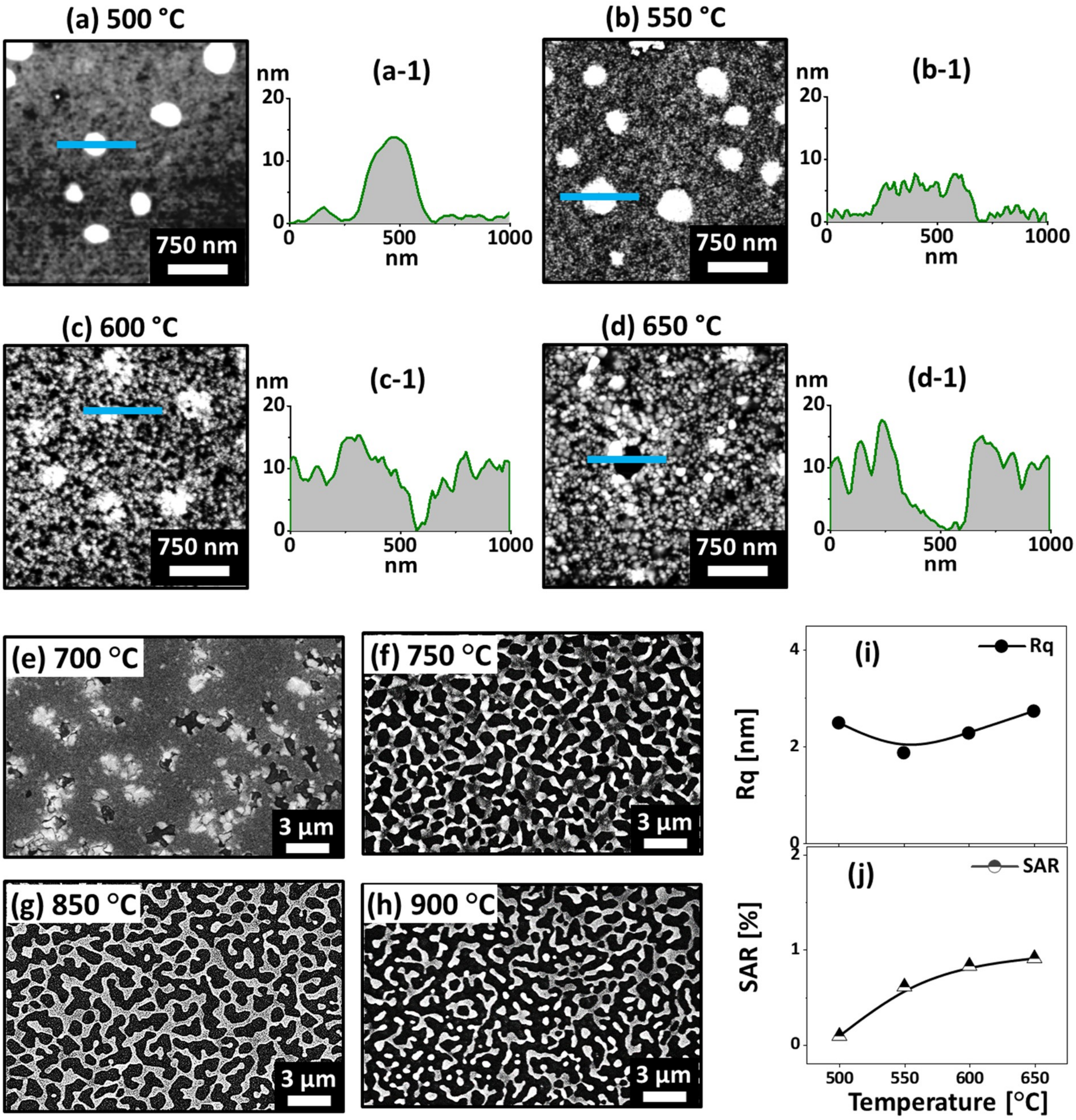

Figure S5: (a) – (d) AFM top-views (3 × 3 μm²) showing the surface morphology of In$_{30\ nm}$ / Pt$_{30\ nm}$ bilayers annealed between 500 and 650 °C. (a-1) – (d-1) Cross-sectional line-profiles. (e) – (h) Scanning electron microscopy (SEM) images. (i) – (j) Plots of Rq and SAR.

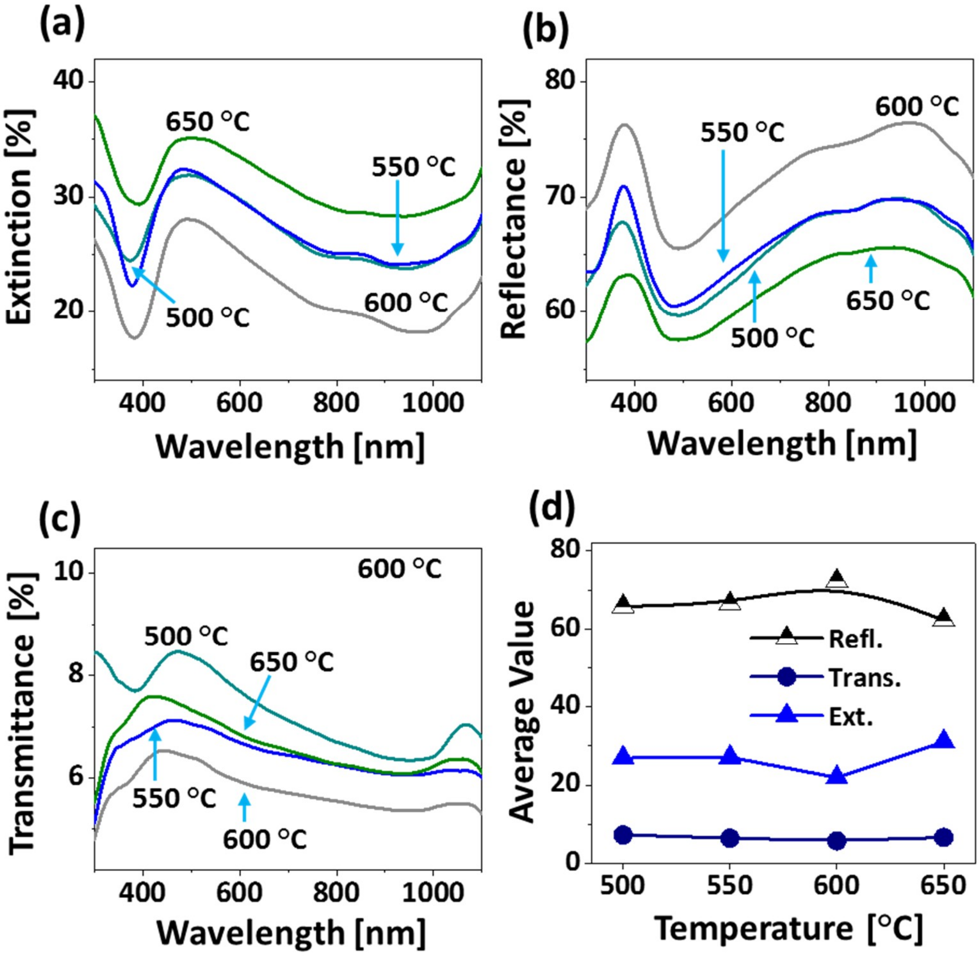

Figure S6: Optical properties of Pt nanostructures fabricated with the $In_{30\,nm}$ / $Pt_{30\,nm}$ bilayers. (a) Extinction, (b) reflectance and (c) transmittance spectra. (d) Plots of average value of reflectance, transmittance, and extinction.

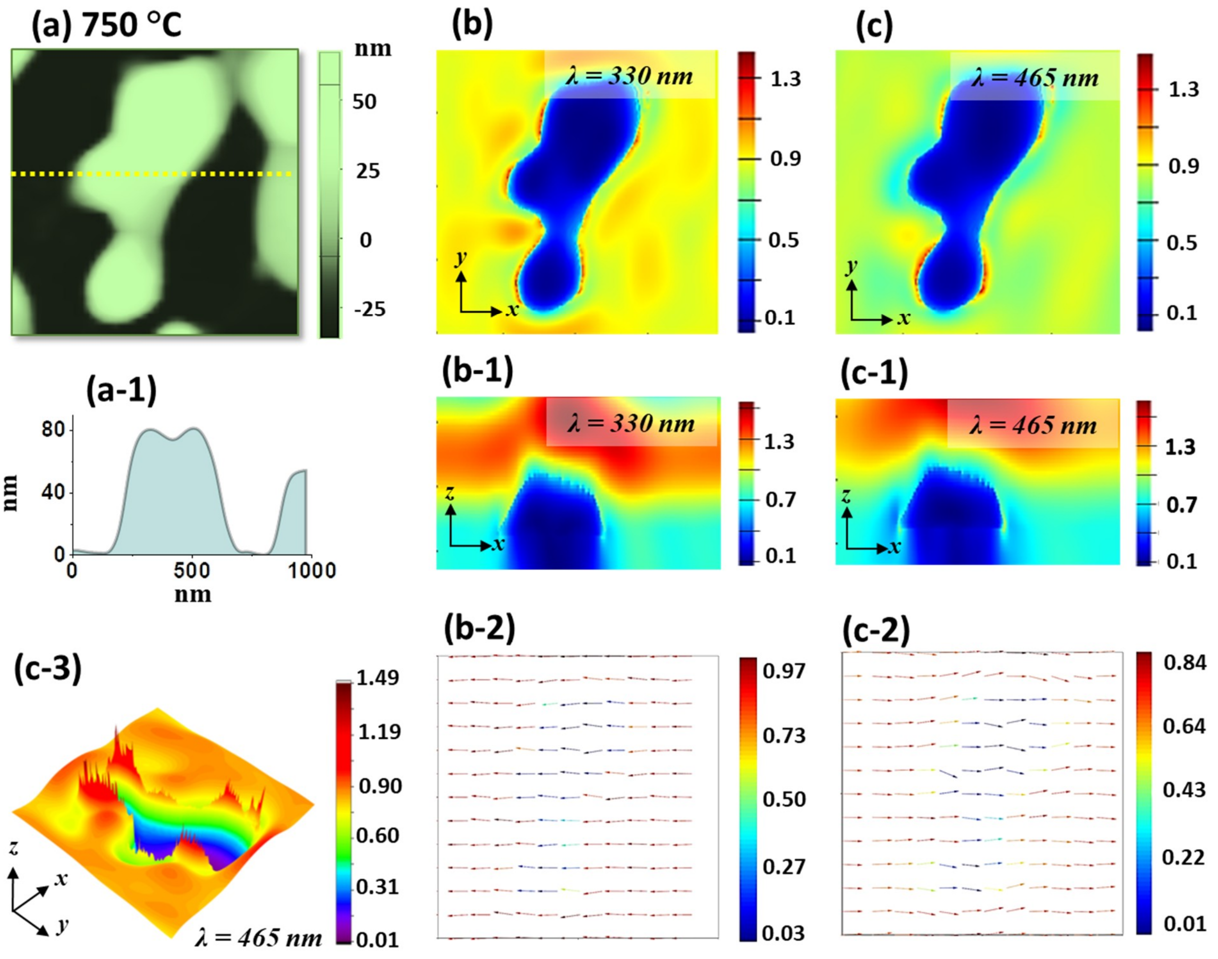

Figure S7: (a) AFM image of typical Pt NPs fabricated at 750 °C with the $In_{30\ nm}$ / $Pt_{30\ nm}$. (a-1) Cross-sectional line-profiles. (b) – (c) Electric (E)-field profiles of NPs in xy-plane at different wavelengths. (b-1) – (c-1) E-field profiles in xz-plane. (b-2) – (c-2) E-field vector plots in xy-plane. (c-3) 3D-view of the e-field distribution.

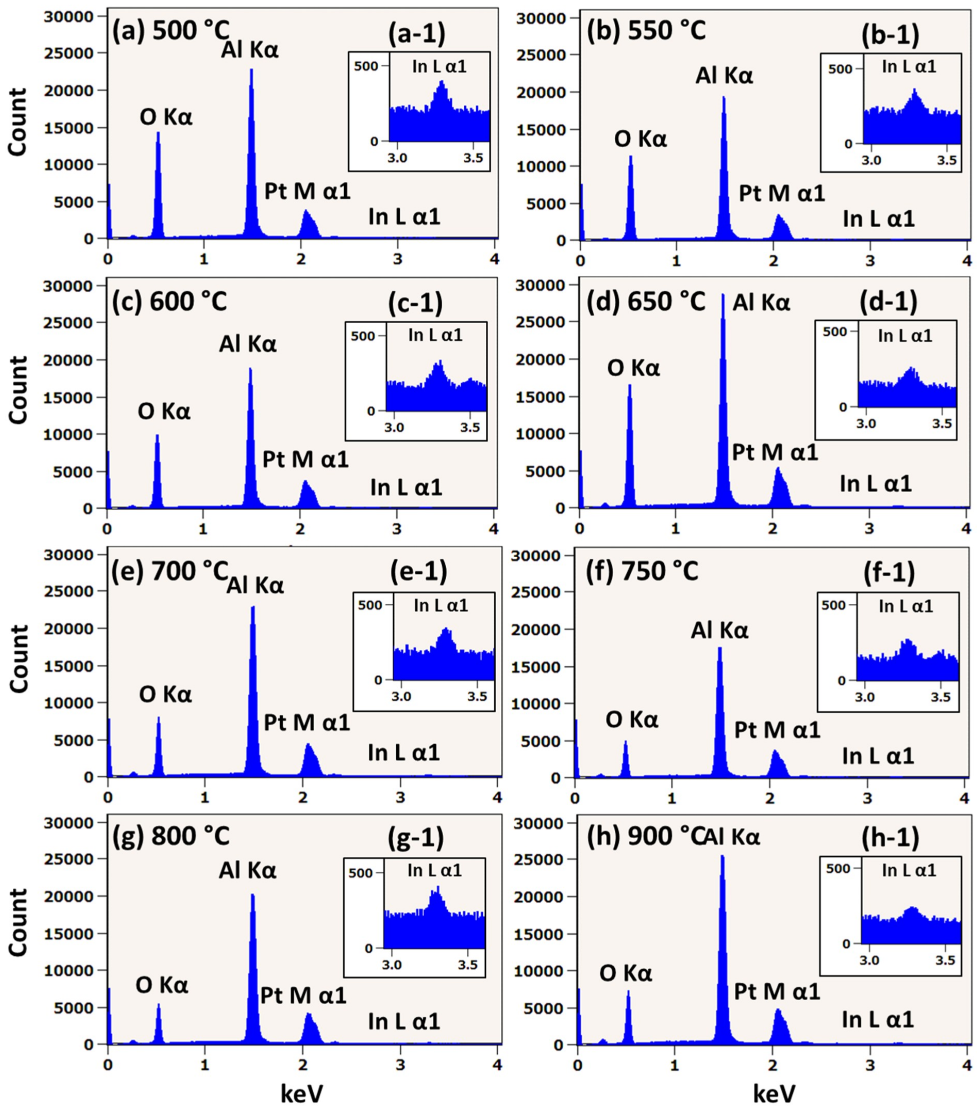

Figure S9: (a) – (h) EDS spectra of various Pt nanostructures morphology on sapphire with the $In_{30\ nm}$ / $Pt_{30\ nm}$ bilayers annealed between 500 and 900 °C for 450 s.

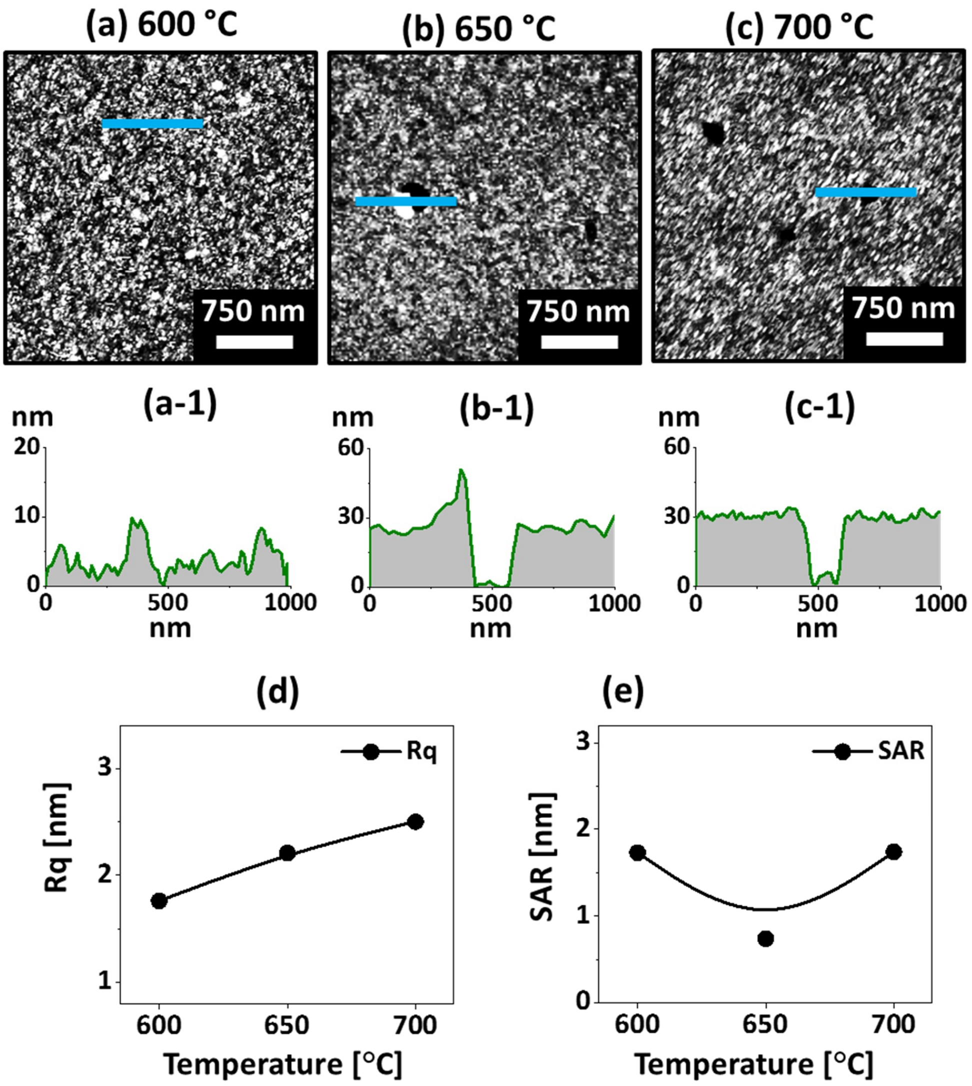

Figure S9: Surface morphology evolution of the $In_{10\ nm}$ / $Pt_{30\ nm}$ bilayers at low annealing temperature between 600 and 700 °C. (a) – (c) AFM top-views (3 × 3 μm²) and (a-1) – (c-1) cross-sectional line-profiles. (d) – (e) Plots of Rq and SAR.

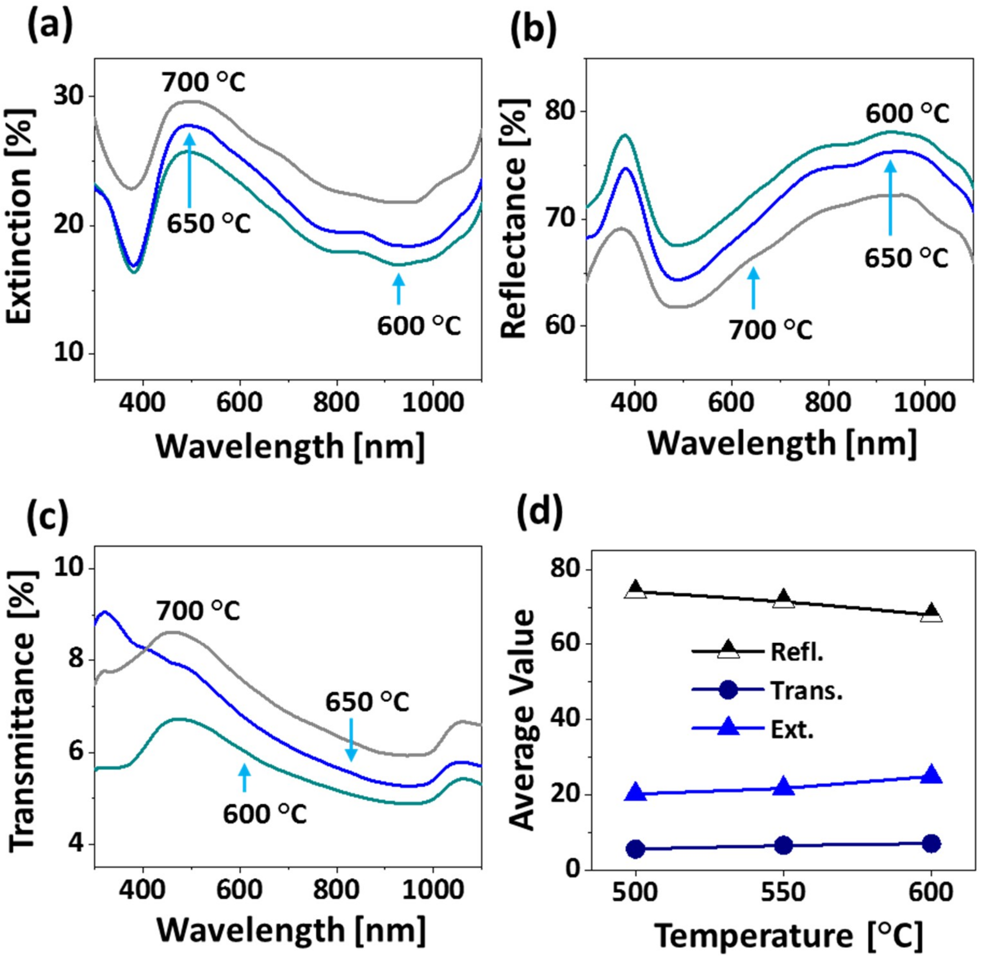

Figure S10: Optical properties of Pt nanostructures fabricated with the $In_{10\,nm}$ / $Pt_{30\,nm}$ bilayers. (a) Extinction, (b) reflectance, (c) transmittance and (d) plot of average value of reflectance, transmittance, and extinction.

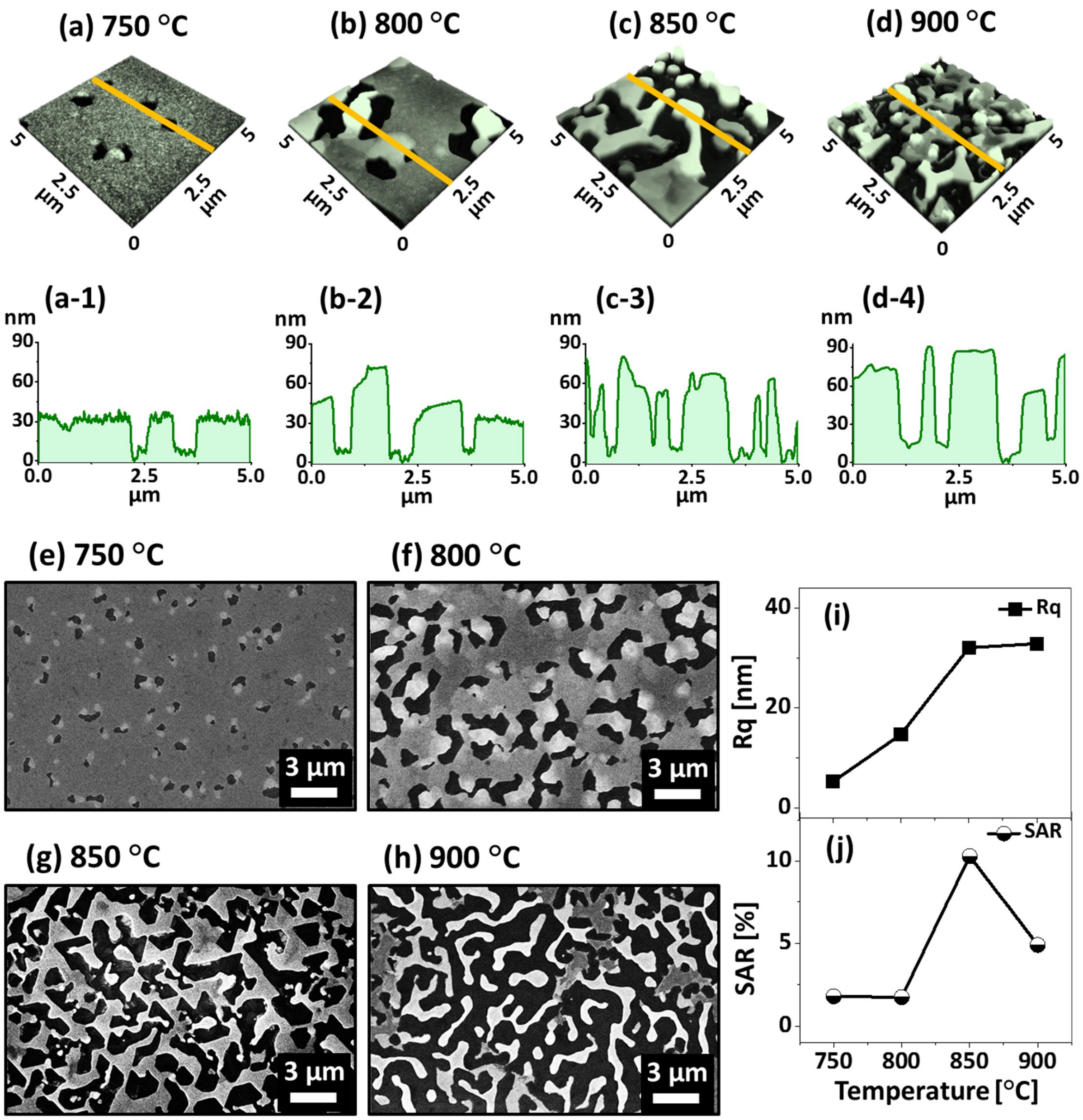

Figure S11: Growth of connected Pt nanostructures along with the increased annealing temperature between 750 and 900 °C for 450 s. The individual film thickness of In and Pt were 10 and 30 nm ($In_{10\,nm}$ / $Pt_{30\,nm}$). (a) – (d) AFM-side views (5 × 5 µm^2). (a-1) – (d-1) Cross-sectional line-profiles. (e) – (h) Corresponding SEM images of various Pt nanostructures. (i) – (j) Plots of Rq and SAR.

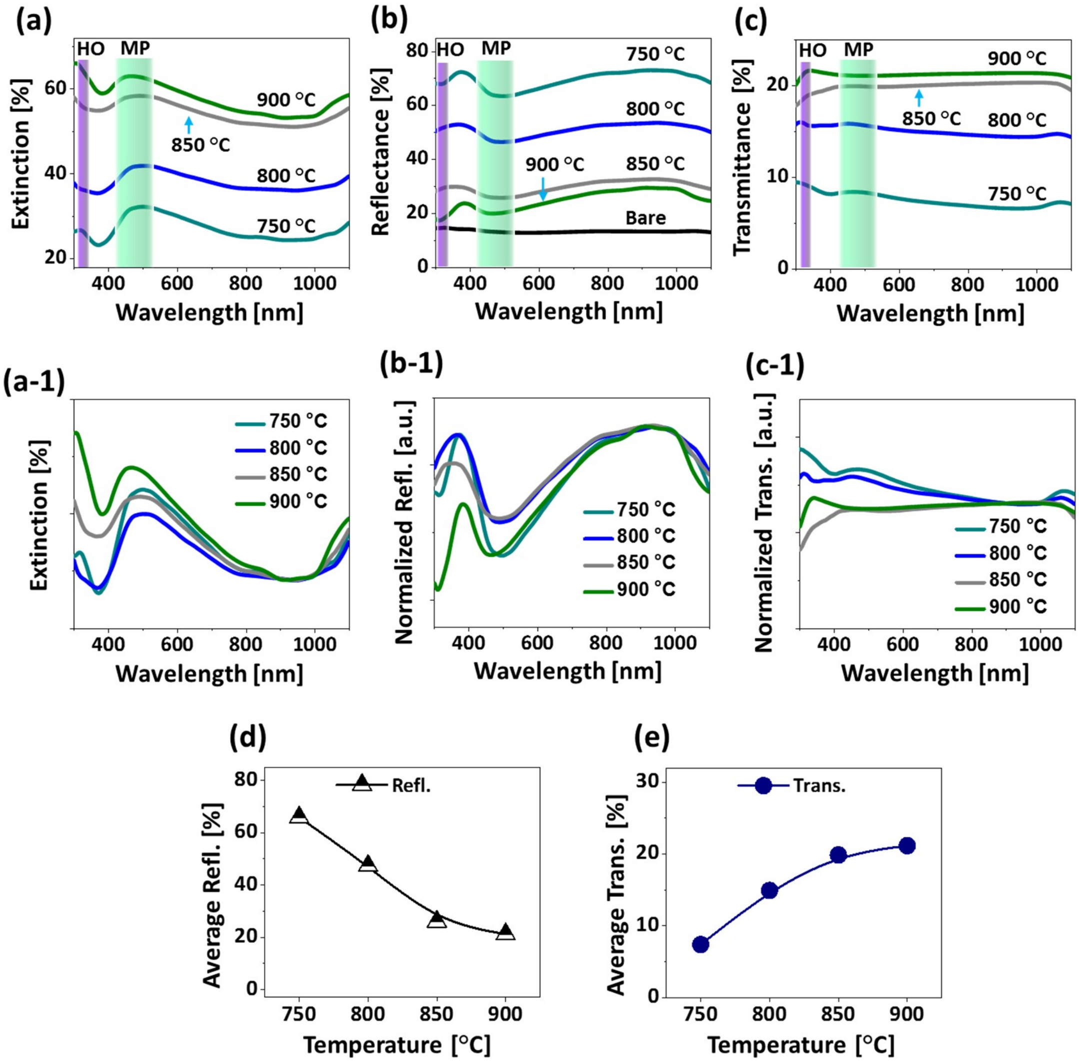

Figure S12: Extinction, reflectance and transmittance spectra of various Pt nanostructures fabricated with the $In_{10\ nm}$ / $Pt_{30\ nm}$ bilayers and annealing between 750 and 900 ^{o}C. (a) Extinction spectra. (a-1) Normalized extinction spectra. (b) Reflectance spectra. (b-1) Normalized reflectance spectra. (c) Transmittance spectra. (c-1) Normalized transmittance spectra. (d) – (e) Plots of average reflectance and transmittance.

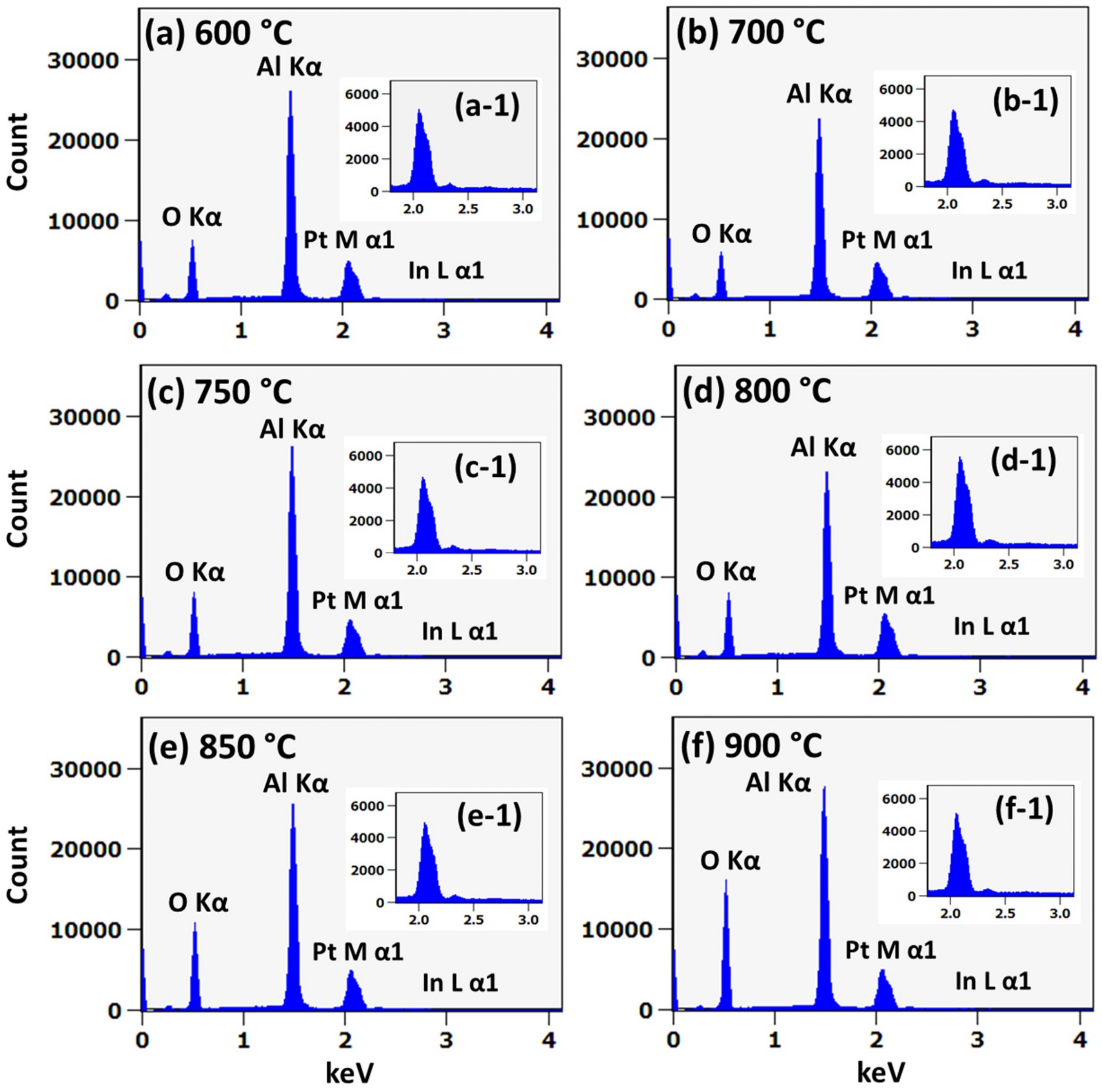

Figure S13: (a) – (f) EDS spectra of Pt nanostructures on sapphire with the $In_{10\ nm}$ / $Pt_{30\ nm}$ bilayers after annealing at various temperature between 600 and 900 ^{o}C.

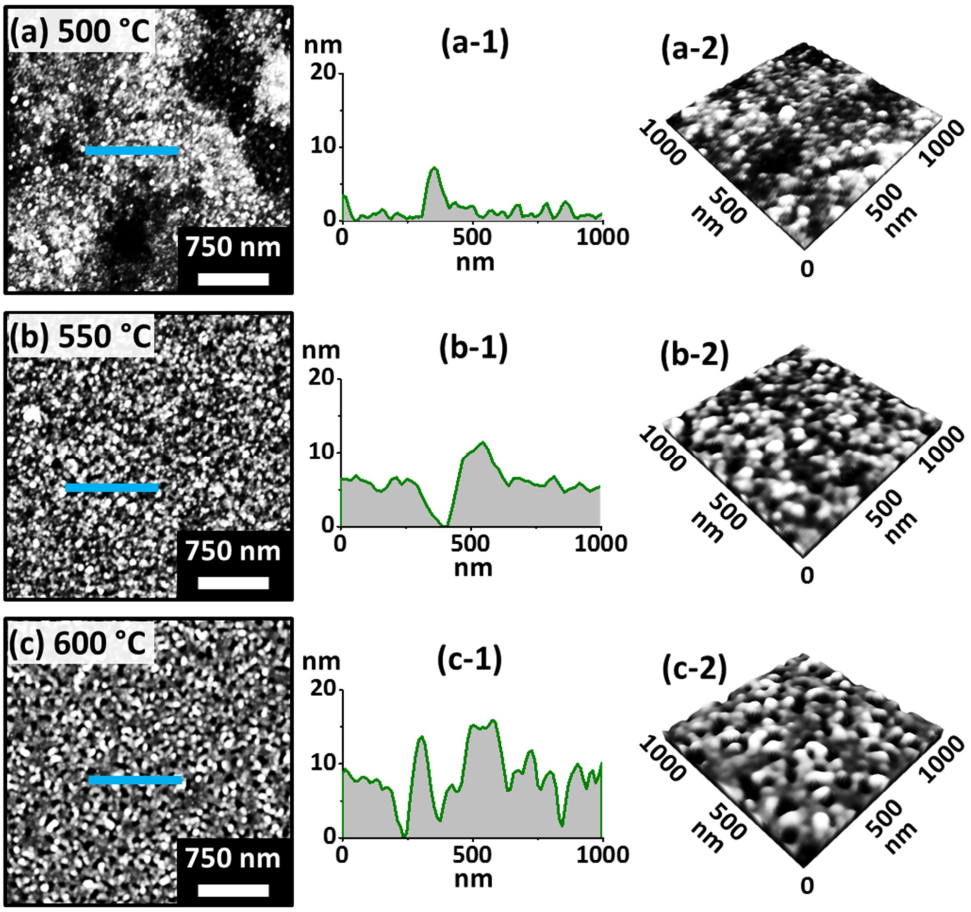

Figure S14: Low temperature dewetting behavior of In_{45nm} / Pt_{15nm} bilayers deposited on sapphire. (a) – (c) AFM top-views (3 × 3 μm²) of samples annealed between 500 and 600 °C for 450 s. (a-1) – (c-1) Cross-sectional line-profiles. (a-2) – (c-2) Magnified AFM side-views (1 × 1 μm²).

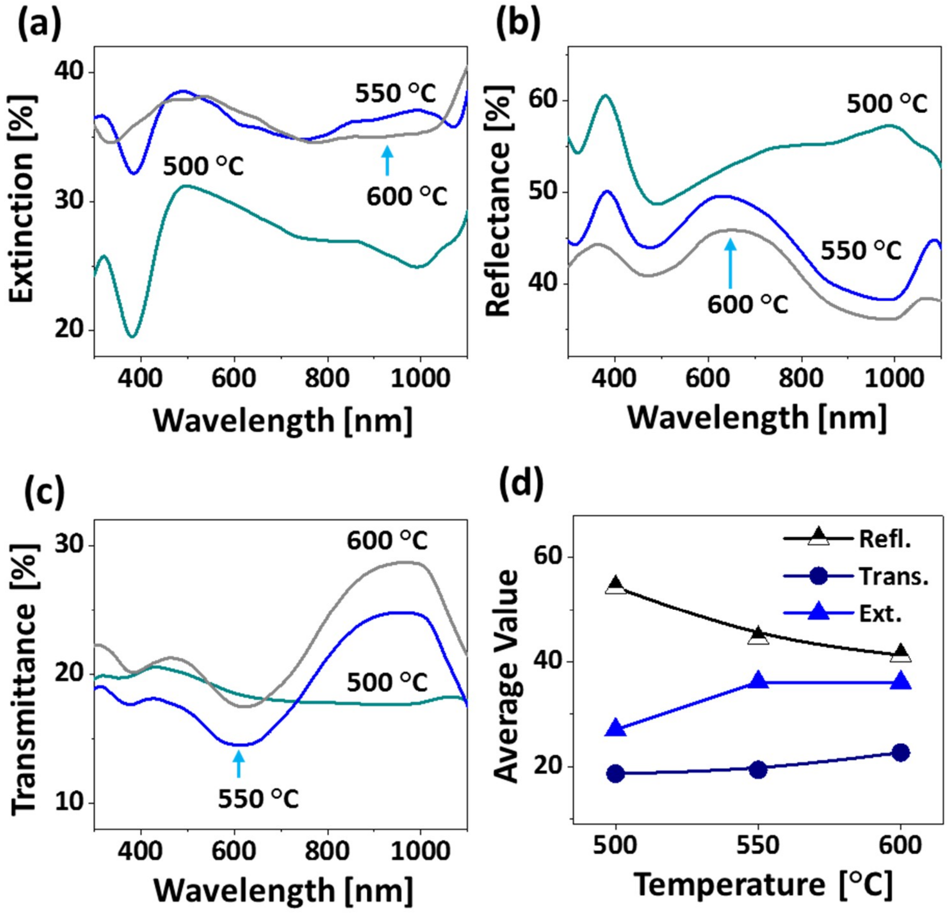

Figure S15: Optical properties of Pt nanostructures fabricated with the In_{45nm} / Pt_{15nm} bilayers at different annealing temperature. (a) Extinction, (b) reflectance and (c) transmittance spectra. (d) Plot of average value of reflectance (Refl.), transmittance (Trans.), and extinction (Ext.).

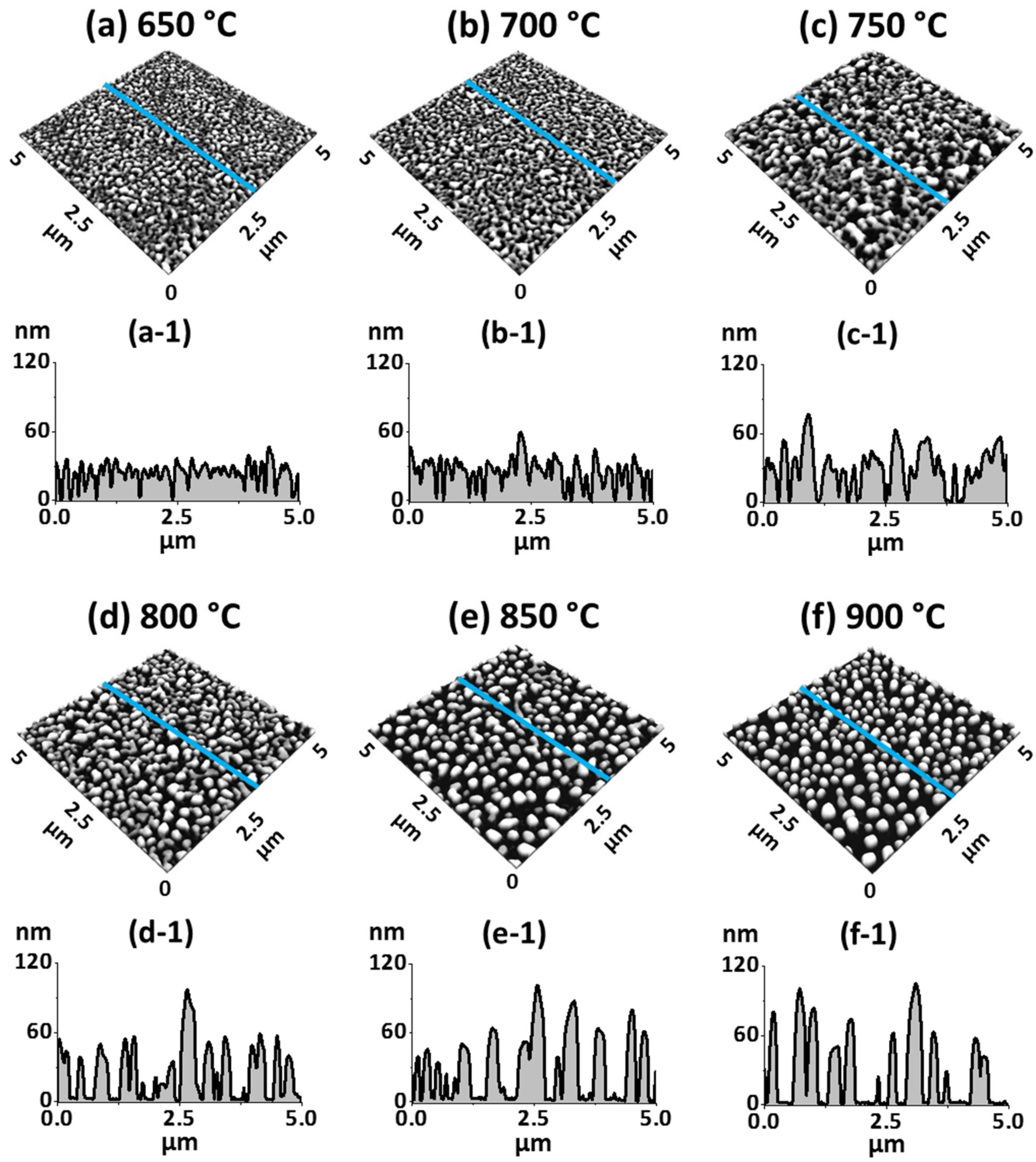

Figure S16: Fabrication of various morphologies of Pt nanostructures by the annealing of $In_{45\ nm}$ / $Pt_{15\ nm}$ bilayers at various temperature between 650 and 900 °C. (a) – (f) AFM side-views (5 × 5 µm^2). (a-1) – (f-1) Corresponding cross-sectional line-profiles.

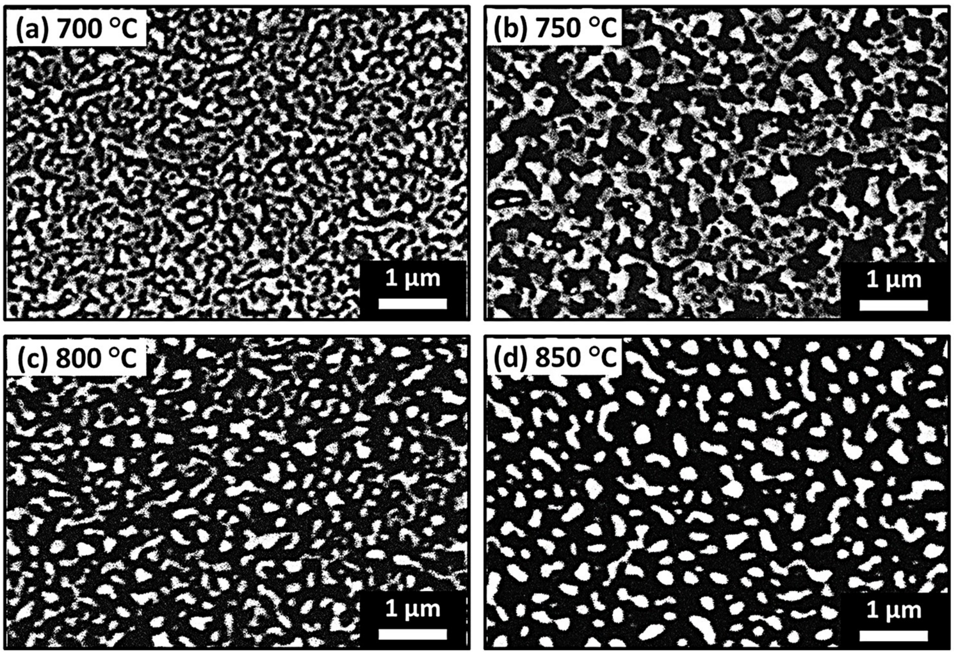

Figure S17: (a) – (d) SEM images of various Pt nanostructures fabricated with the $In_{45\ nm}$ / $Pt_{15\ nm}$ bilayers at various temperature between 650 and 900 °C for 450 s.

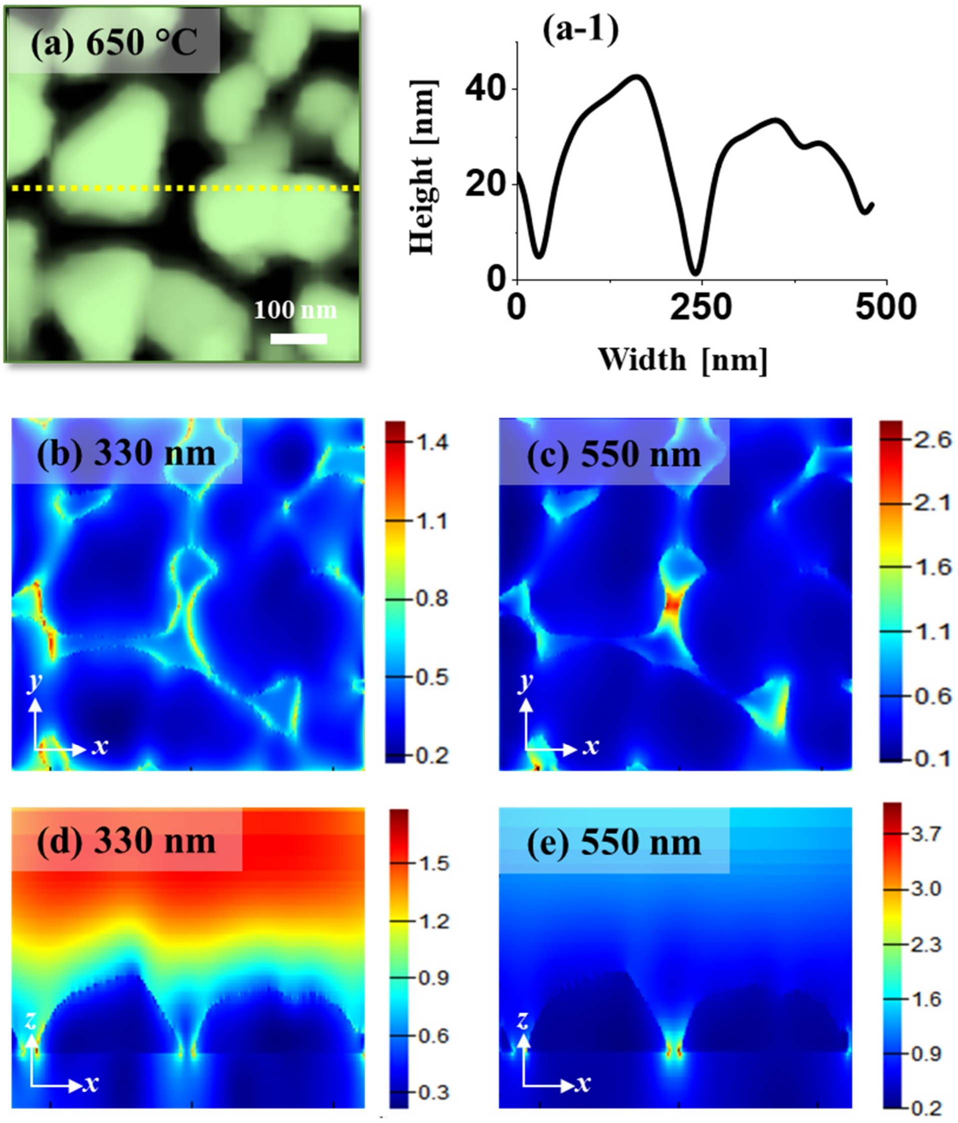

Figure S18: Finite difference time domain (FDTD) simulation of the Pt NPs fabricated at 650 °C with the $In_{45\,nm}$ / $Pt_{15\,nm}$ bilayers. (a) AFM image. (a-1) Cross-sectional line profile. (b) – (c) E-field profiles in xy-plane. (d) – (e) E-field profiles in xz-plane.

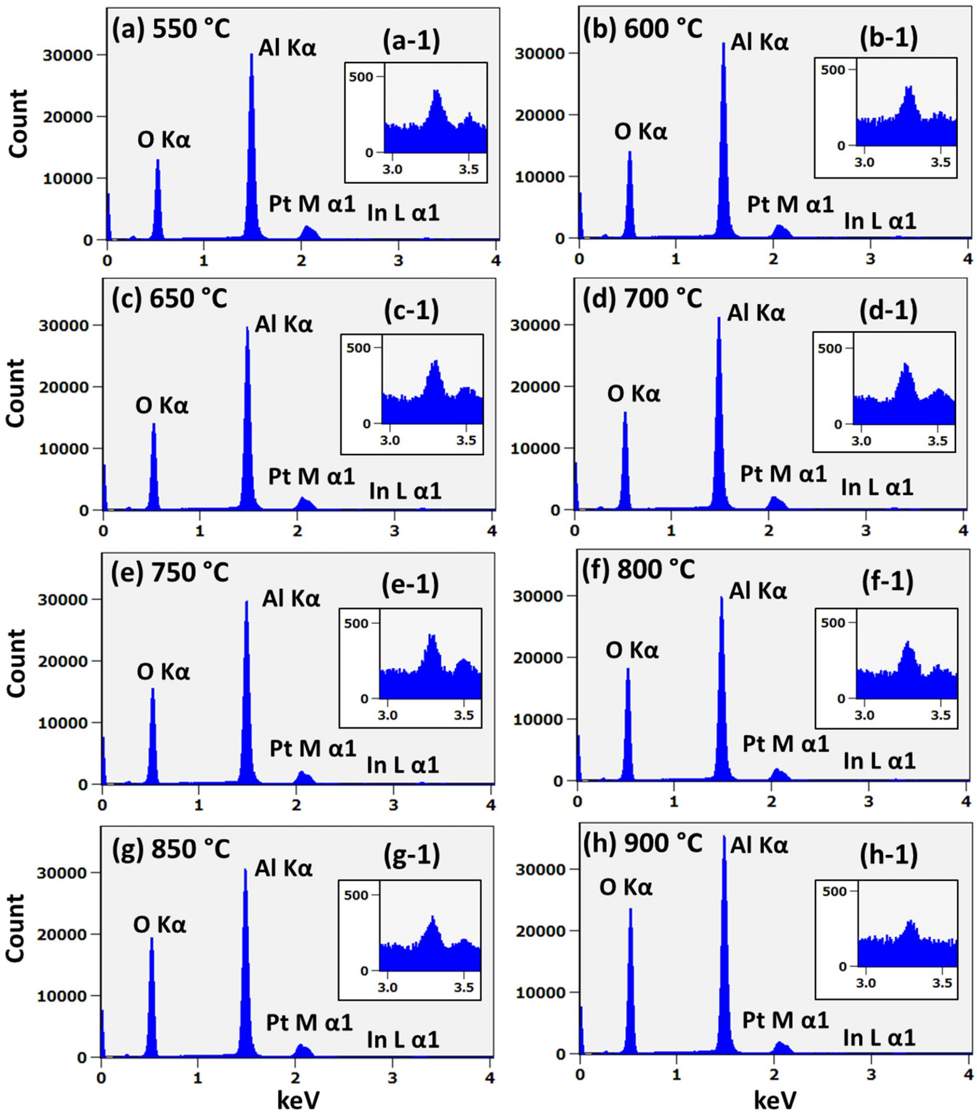

Figure S19: EDS spectra of various Pt nanostructures on sapphire (0001) fabricated with the $In_{45\,nm}$ / Pt $_{15\,nm}$ bilayers by annealing temperature between 550 and 900 ^{o}C.

Table S1: Summary of Rq and SAR of various Pt nanostructures on sapphire (0001) with various In / Pt bilayer thickness and annealing temperature.

**Temperature [°C]**	**In $_{45\ nm}$ / Pt $_{15\ nm}$**		**In $_{30\ nm}$ / Pt $_{30\ nm}$**	
	**Rq [nm]**	**SAR [%]**	**Rq [nm]**	**SAR [%]**
**500**	1.16	0.22	2.49	0.09
**550**	1.52	0.54	1.87	0.61
**600**	3.52	2.54	2.27	0.83
**650**	9.07	8.73	2.73	0.91
**700**	12.01	11.01	8.62	1.25
**750**	16.82	12.15	31.52	6.07
**800**	22.38	20.66	-	-
**850**	26.85	18.82	34.33	9.41
**900**	28.18	21.33	34.45	7.42

Table S2: Summary of Rq and SAR of various Pt nanostructures on sapphire (0001) with various In / Pt bilayer thickness followed by the annealing temperature.

**Temperature [°C]**	**In $_{10\ nm}$ / Pt $_{30\ nm}$**		**In $_{2\ nm}$ / Pt $_{2\ nm}$**	
	**Rq [nm]**	**SAR [%]**	**Rq [nm]**	**SAR [%]**
**500**	-	-	-	-
**550**	-	-	1.72	5.51
**600**	1.76	1.73	-	-
**650**	2.21	0.74	1.85	5.31
**700**	2.50	1.74	1.87	5.37
**750**	5.24	1.79	2.15	6.48
**800**	14.65	1.74	-	-
**850**	32.08	10.30	-	-
**900**	32.90	4.90	-	-

Table S3: Summary of average reflectance, transmittance and extinction of various Pt nanostructures on sapphire (0001) with various In / Pt bilayer thickness and annealing temperature.

Temperature [°C]	In 45 nm / Pt 15 nm		In 30 nm / Pt 30 nm	
	**Refl.**	**Trans.**	**Refl.**	**Trans.**
**Bare**	12.98	86.37	12.98	86.37
**500**	54.35	18.63	65.78	7.29
**550**	44.54	19.35	66.44	6.44
**600**	41.25	22.66	72.30	5.76
**650**	34.91	29.89	62.27	6.63
**700**	32.16	32.67	53.87	11.51
**750**	28.52	35.83	28.71	29.07
**800**	24.39	40.51	-	-
**850**	21.38	44.78	20.90	32.78
**900**	16.62	48.39	19.63	37.29

Table S4: Summary of average reflectance, transmittance and extinction of various Pt nanostructures fabricated with various In / Pt bilayer thickness at different annealing temperatures.

Temperature [°C]	In $_{10\ nm}$ / Pt $_{30\ nm}$		In $_{2\ nm}$ / Pt $_{2\ nm}$	
	**Refl.**	**Trans.**	**Refl.**	**Trans.**
**Bare**	12.98	86.37	-	-
**500**	-	-	-	-
**550**	-	-	17.27	72.29
**600**	74.10	5.63	-	-
**650**	71.64	6.61	16.82	73.67
**700**	67.94	7.11	15.78	74.03
**750**	65.81	7.38	14.89	75.23
**800**	47.31	14.92	-	-
**850**	25.99	19.86	-	-
**900**	21.16	21.16	-	-
